# Metabolomic Differences Between Wild and Cultivated *Ophiocordyceps sinensis* Identify Source-Associated Metabolites with Predicted Antifibrotic Relevance

**DOI:** 10.3390/ijms27219558

**Published:** 2026-10-26

**Authors:** Meng-Yuan Jin, Jiayi Li, Jun Lan, Bai-La De, Dong-Qing Wei, Li-Da Zhang, Xuan-Wei Zhou

**Affiliations:** 1 School of Agriculture and Biology, Shanghai Jiao Tong University, Shanghai 200240, China; jinmengyuan@sjtu.edu.cn (M.-Y.J.); xuanweizhou@sjtu.edu.cn (X.-W.Z.); 2 State Key Laboratory of Microbial Metabolism, Joint International Research Laboratory of Metabolic & Developmental Sciences and School of Life Sciences and Biotechnology, Shanghai Jiao Tong University, Shanghai 200240, China; jemmylee@sjtu.edu.cn (J.L.); dqwei@sjtu.edu.cn (D.-Q.W.); 3 NMPA Key Laboratory of Quality Control of Traditional Chinese Medicine, Tibetan Medicine, Xizang Institute for Food and Drug Control, Lhasa 850000, China; lanjun1252021@163.com

**Keywords:** *O. sinensis*, untargeted metabolomics, targeted quantification, network pharmacology, molecular dynamics, liver fibrosis

## Abstract

*Ophiocordyceps sinensis* (*O. sinensis*), a precious traditional Chinese medicine (TCM), exhibits remarkable hepatoprotective effects. However, chemical differences between wild-collected and cultivated products remain insufficiently characterized in relation to liver fibrosis. We combined untargeted ultra-high-performance liquid chromatography–Orbitrap high-resolution mass spectrometry with targeted liquid chromatography–tandem mass spectrometry to compare wild type (WT) and artificially cultivated (AC) specimens. The two sample groups showed distinct metabolic profiles, and targeted analysis supported differences in selected nucleosides, purine derivatives, pyroglutamic acid, and ergosterol. Adenosine, cordycepin, and ergosterol were more abundant in AC extracts, whereas hypoxanthine, deoxyguanosine, guanosine, and guanine were more abundant in WT extracts. Network pharmacology associated selected source-related metabolites with 158 fibrosis-related candidate targets and prioritized a kinase-centered network involving epidermal growth factor receptor (EGFR), proto-oncogene tyrosine-protein kinase Src (SRC), protein tyrosine kinase 2 (PTK2), HRas proto-oncogene, GTPase (HRAS), phosphatidylinositol 3-kinase/protein kinase B (PI3K-Akt), and mitogen-activated protein kinase (MAPK) signaling. Molecular docking and replicated molecular dynamics simulations indicated structurally plausible interactions for complexes of uridine diphosphate N-acetylglucosamine (UDP-N-acetylglucosamine) with HRAS and AKT serine/threonine kinase 1 (AKT1), and of ergosterol with SRC. These findings reveal source-associated metabolic differences between the analyzed WT and AC samples and provide mechanistic insights into their potentially divergent roles in liver fibrosis regulation, which warrant further experimental validation.

## 1. Introduction

*Ophiocordyceps sinensis* (*O. sinensis*), a medicinal fungus belonging to the family Ophiocordycipitaceae, parasitizes the larvae of ghost moths and has long been used as a highly valued traditional Chinese medicine (TCM) [1]. *O. sinensis* contains diverse metabolites with reported immunomodulatory, antioxidant, metabolic, anti-fibrotic, and other biological activities [2,3]. However, wild type (WT) *O. sinensis* resources have declined markedly because of ecological disturbance, intensive harvesting, its long life cycle, and limited natural regeneration [4]. Artificially cultivated (AC) *O. sinensis* and the development of other *Cordyceps*-derived products have therefore provided alternative resources and helped reduce harvesting pressure on wild populations [5,6].

The chemical composition of *O. sinensis* is influenced by its growth environment. Wild and cultivated samples develop under substantially different ecological conditions, particularly with respect to host-associated and environmental microbial communities [7,8,9]. These differences may affect metabolic processes and, consequently, metabolite accumulation. Several major constituents of AC occur at levels comparable to those found in WT, supporting its potential use as an alternative medicinal resource [10,11]. However, similarity in a limited number of marker compounds does not establish equivalence in overall chemical composition or pharmacological activity. A broader comparison of the biologically relevant chemical profiles of WT and AC is therefore required.

Liver fibrosis is a pathological wound-healing response to chronic liver injury and is characterized by persistent inflammation, hepatic stellate cell (HSC) activation, and excessive extracellular matrix deposition and remodeling [12]. Previous clinical and experimental investigations provide a rationale for exploring the potential antifibrotic relevance of *O. sinensis*. A preliminary clinical study reported improvements in serum markers of fibrosis in patients with chronic hepatitis B [13]. Experimental studies reported that *O. sinensis* alleviated Carbon tetrachloride (CCl_4_)-plus-ethanol-induced hepatic fibrosis in rats [14]. Polysaccharides isolated from cultured *O. sinensis* mycelia were subsequently shown to attenuate CCl_4_-induced hepatic fibrosis, accompanied by reduced HSC activation and modulation of transforming growth factor beta 1 (TGF-β1)/Smad signaling [15]. Furthermore, cultured *O. sinensis* mycelium reduced inflammation and fibrosis-associated changes in thioacemide-induced rat liver injury [16]. Importantly, ergosterol isolated from cultured mycelia exhibited antifibrotic activity in CCl4-treated mice and activated LX-2 cells, a human hepatic stellate cell line, providing evidence for the contribution of a specific small-molecule constituent [17]. These findings support the selection of liver fibrosis as a biologically relevant context for investigating *O. sinensis*-derived metabolites. Nevertheless, previous studies have primarily focused on the pharmacological activities of *O. sinensis*-derived preparations or individual constituents, while the potential implications of compositional differences between wild and artificially cultivated *O. sinensis* remain insufficiently understood. In particular, whether their distinct metabolite profiles correspond to different sets of candidate antifibrotic constituents and associated molecular target–pathway interactions has not been systematically investigated.

Metabolomics provides a comprehensive approach for characterizing chemical variation in medicinal materials and has been widely applied to investigate compositional differences associated with geographical origin, cultivation conditions, and biological sources [18,19,20]. Network pharmacology integrates compound-, target-, and disease-related information to prioritize candidate metabolites, molecular targets, and signaling pathways for subsequent biological investigation [21]. Molecular dynamics (MD) simulations can further characterize the time-dependent conformational behavior and interaction stability of selected metabolite-target complexes, thereby generating structure-based hypotheses for experimental validation [22]. Together, these complementary approaches offer a framework for linking source-associated chemical differences to potentially distinct antifibrotic constituent profiles and predicted molecular interaction features, generating hypotheses for subsequent experimental validation.

In this study, ultra-high-performance liquid chromatography–Orbitrap high-resolution mass spectrometry (UHPLC–Orbitrap HRMS)-based untargeted metabolomics was first used to characterize source-associated metabolic profiles and identify metabolites differing between WT and AC. Selected metabolites were subsequently quantified by ultra-performance liquid chromatography–tandem mass spectrometry (UPLC–MS/MS) in an independent sample set to assess key compositional differences. Candidate metabolites associated with the two sources were then investigated using network pharmacology and functional enrichment analyses within the context of liver fibrosis. Representative metabolite–target pairs were further examined by molecular docking and molecular dynamics simulations. This stepwise strategy was designed to trace source-associated metabolite variation through predicted targets and pathways to specific structural interactions, thereby generating testable hypotheses regarding potential differences in the antifibrotic chemical basis of WT and AC *O. sinensis*.

## 2. Results

### 2.1. UHPLC-Orbitrap HRMS Profiling Reveals Distinct Chemical Differences Between AC and WT O. sinensis

**Figure 1:**
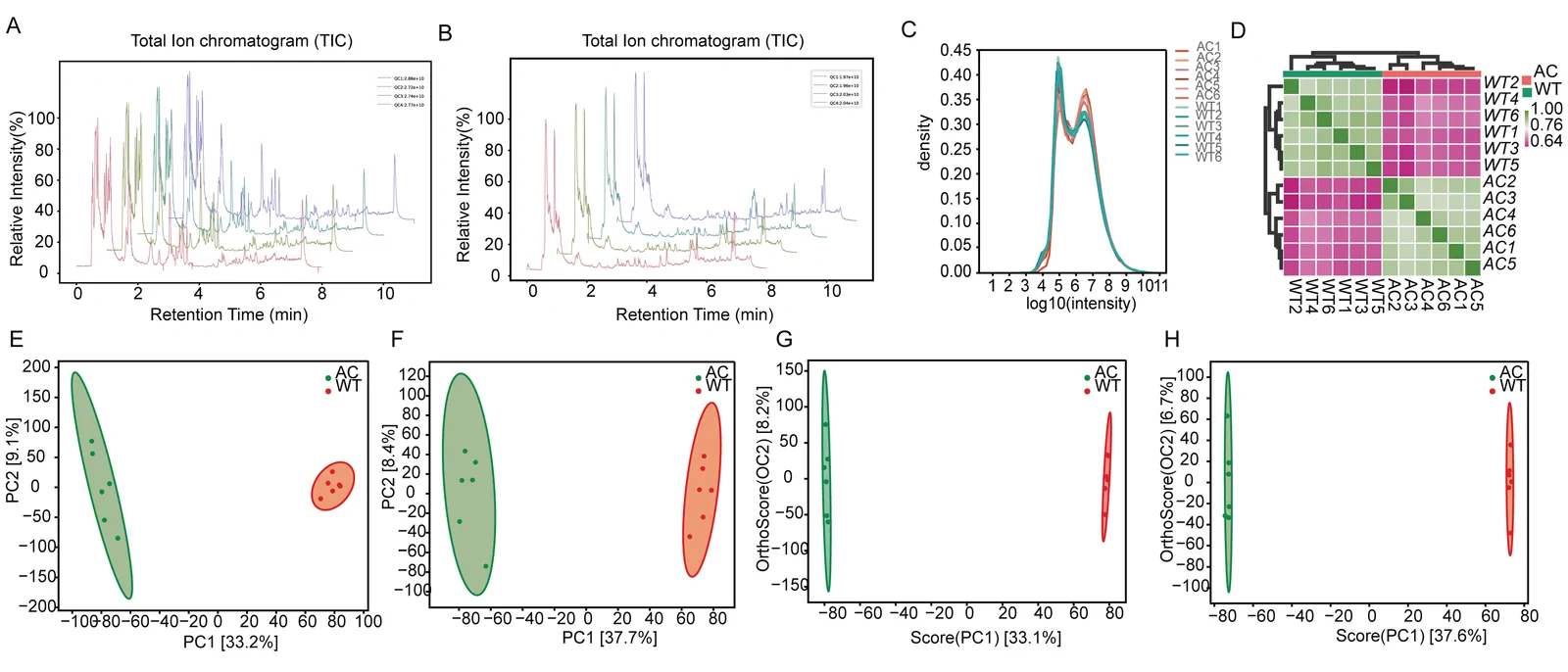
UHPLC-Orbitrap HRMS data quality assessment and multivariate analysis of AC and WT samples. (**A**,**B**) Overlaid TICs of QC samples in positive and negative ion modes, respectively. (**C**) Density distribution of metabolite signal intensities. (**D**) Pearson correlation heatmap in positive ion mode, with green indicating higher and magenta indicating lower sample correlations. (**E**,**F**) PCA score plots and (**G**,**H**) OPLS-DA score plots in positive and negative ion modes, respectively. Untargeted metabolomics included six independent biological replicates per group, with each replicate representing material from one individual specimen.

To evaluate the reliability of the UHPLC-Orbitrap HRMS metabolomic data and compare the global metabolic profiles between WT and AC samples, quality control assessment and multivariate statistical analyses were first performed. The overlaid total ion chromatograms (TICs) of quality control (QC) samples in both positive and negative ion modes showed highly consistent peak retention times and similar peak distribution patterns, indicating good stability of the UHPLC-Orbitrap HRMS system and reproducibility of data acquisition (Figure 1A,B). In addition, the density distribution curves of sample abundance showed generally similar distribution patterns among AC and WT samples (Figure 1C and Supplementary Table S1).

Pearson correlation analysis showed high reproducibility among biological replicates within each group, and AC and WT samples formed distinct clusters in both positive and negative ion modes (Figure 1D and Supplementary Figure S1A). Consistently, principal component analysis (PCA), partial least squares-discriminant analysis (PLS-DA), and orthogonal partial least squares-discriminant analysis (OPLS-DA) score plots all showed clear separation between AC and WT samples, while samples within the same group clustered closely (Figure 1E–H and Supplementary Figure S1B,C). For the AC-versus-WT comparison, the PLS-DA output reported two components and yielded R2X = 0.413, R2Y = 1.000, and Q2 = 0.967, whereas the OPLS-DA model comprised one predictive and one orthogonal component and yielded R2X = 0.413, R2Y = 1.000, and Q2 = 0.947. To further assess potential model overfitting, a permutation test involving 100 random permutations was performed (Supplementary Figure S1D,E). The Q^2^ value of the original model (0.947) exceeded those of all 100 permuted models, for which the maximum Q^2^ was 0.605. The extrapolated Q^2^ intercept was −0.110, and none of the permuted models achieved a Q^2^ value comparable to that of the original model (0/100 permutations; empirical *p* ≈ 0.0099). These results indicate that WT and AC *O. sinensis* exhibited distinct and stable metabolomic profiles.

### 2.2. Differential Metabolite Profiling Reveals Substantial Chemical Differences Between AC and WT O. sinensis

**Figure 2:**
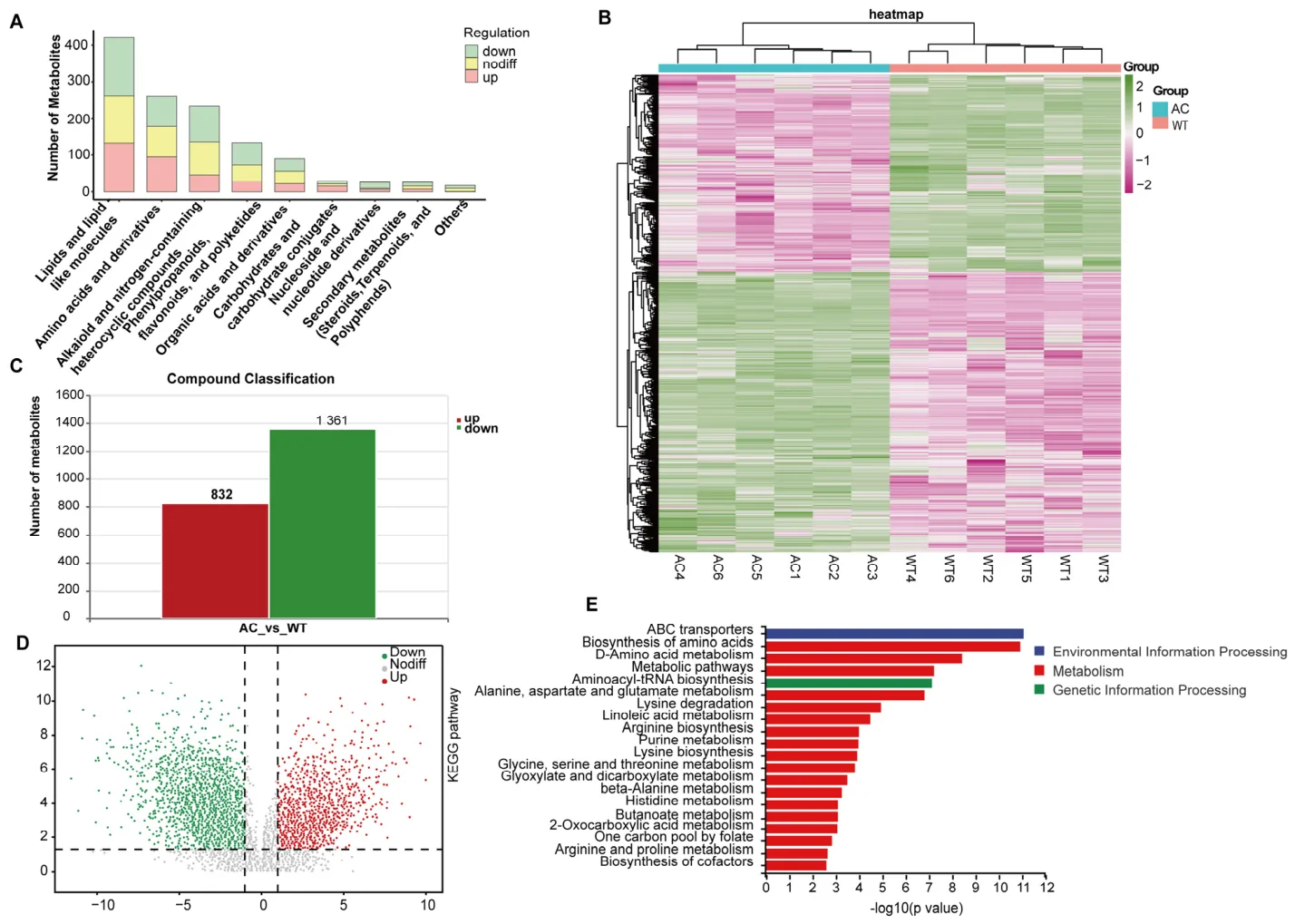
Differential metabolite profiling and pathway enrichment analysis between AC and WT *O. sinensis*. (**A**) Compound classification of differential metabolites in the AC relative to WT. Red, yellow, and green indicate up-regulated, non-regulated, or down-regulated metabolites in AC relative to WT. (**B**) Heatmap of differential metabolites, with green indicating higher and magenta indicating lower abundance. (**C**) Count distribution of differential metabolites in the AC relative to WT. Red represents metabolites up-regulated in AC relative to WT, and green represents metabolites down-regulated in AC relative to WT. (**D**) Volcano plot of differential metabolites between AC and WT samples. Red dots indicate up-regulated metabolites in AC, green dots indicate down-regulated metabolites in AC, and gray dots indicate non-differential metabolites. (**E**) KEGG pathway annotation of differential metabolites.

Based on the global metabolomic differences observed between cultivated and wild *O. sinensis*, differential metabolites were further screened between the AC and WT groups. Compound classification analysis showed that the detected metabolites were mainly distributed in lipids and lipid-like molecules, amino acid derivatives, alkaloids and nitrogen-containing heterocyclic compounds, phenylpropanoids, polyketides, flavonoids, organic acids and derivatives, carbohydrates and carbohydrate conjugates, and nucleosides and nucleotide derivatives (Figure 2A). Among these categories, lipids and lipid-like molecules represented the largest class, followed by amino acid derivatives and alkaloid-related compounds, suggesting that lipid metabolism and amino acid-related metabolism may be important sources of chemical variation between WT and AC samples.

The heatmap analysis of the differential metabolites showed a clear separation between AC and WT samples. The biological replicates from each group clustered together, and the differential metabolites formed distinct expression patterns between the two groups, indicating good intra-group consistency and marked metabolic differences between AC and WT samples (Figure 2B). Quantitative analysis identified a total of 2193 differential metabolites in the AC and WT comparison, including 832 up-regulated and 1361 down-regulated metabolites in AC relative to WT (Figure 2C). Consistently, the volcano plot showed a broad distribution of significantly changed metabolites, with more down-regulated than up-regulated metabolites in AC compared with WT samples (Figure 2D).

To further explore the biological relevance of these differential metabolites, Kyoto Encyclopedia of Genes and Genomes (KEGG) pathway enrichment analysis was performed. The differential metabolites were mainly enriched in adenosine triphosphate (ATP)-binding cassette (ABC) transporters, biosynthesis of amino acids, D-amino acid metabolism, metabolic pathways, aminoacyl-tRNA (transfer ribonucleic acid) biosynthesis, alanine, aspartate and glutamate metabolism, lysine degradation, linoleic acid metabolism, arginine biosynthesis, purine metabolism, lysine biosynthesis, glycine, serine and threonine metabolism, and glyoxylate and dicarboxylate metabolism (Figure 2E). These enriched pathways were primarily associated with metabolism, environmental information processing, and genetic information processing. Together, these results indicate that AC and WT *O. sinensis* differ substantially in their metabolite composition, especially in lipid-related metabolites, amino acid derivatives, and nucleotide-related metabolic pathways.

### 2.3. Identification and Quantitative Validation of Pharmacologically Active Metabolites in O. sinensis

**Figure 3:**
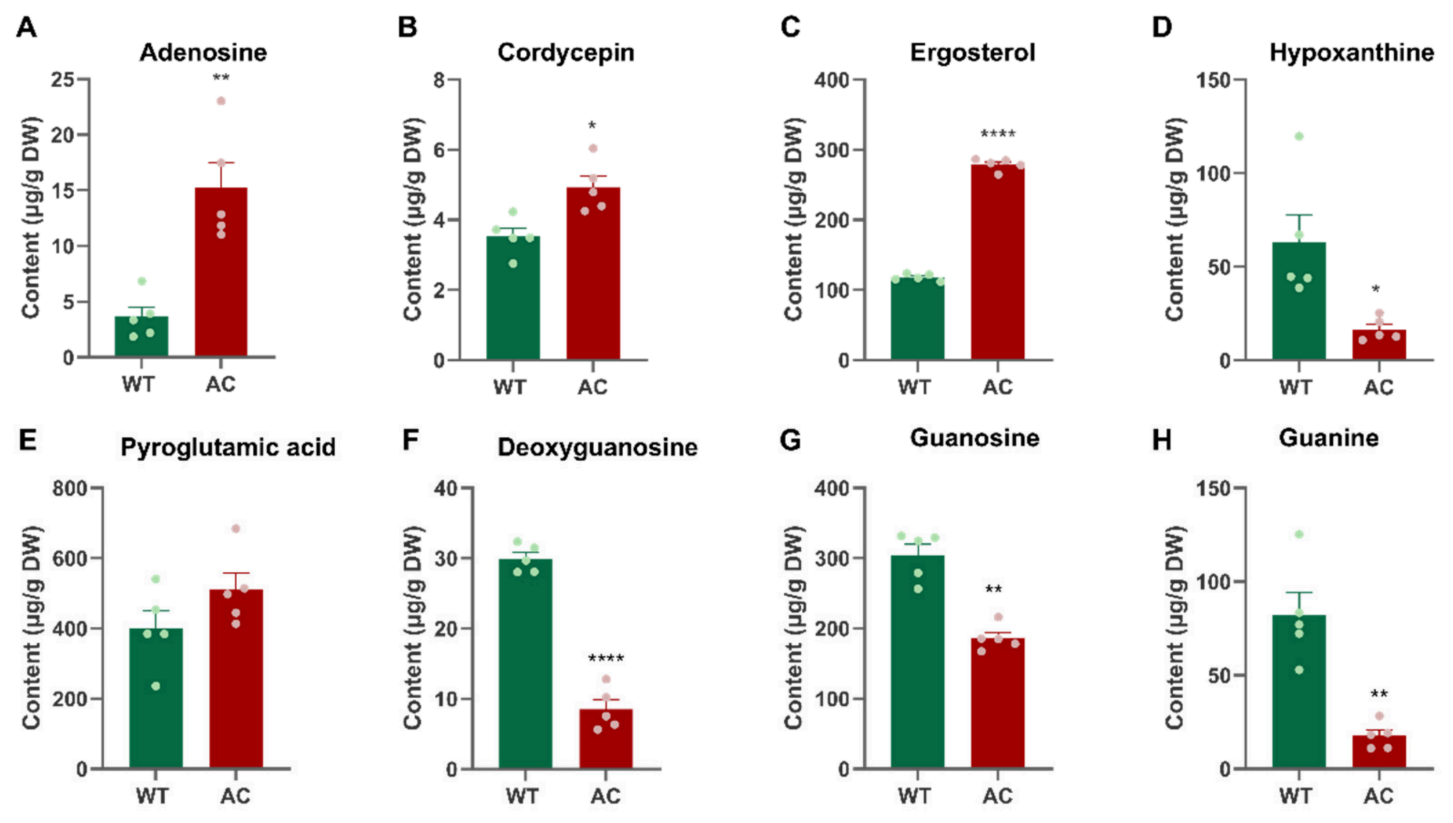
Targeted quantification of selected metabolites in WT and AC *O. sinensis*. The contents of adenosine (**A**), cordycepin (**B**), ergosterol (**C**), hypoxanthine (**D**), pyroglutamic acid (**E**), deoxyguanosine (**F**), guanosine (**G**), and guanine (**H**) in WT and AC *O. sinensis* samples were determined by LC–MS/MS using external calibration curves established with authentic reference standards. Data are presented as mean ± SEM (*n* = 5 biological replicates per group). Statistical significance was determined using two-tailed unpaired Welch’s *t*-tests followed by Benjamini–Hochberg false discovery rate (FDR) correction across eight metabolites. * q < 0.05, ** q < 0.01, **** q < 0.0001; ns, not significant. DW: dry weight.

To further characterize the differences in selected metabolites between WT and AC *O. sinensis*, eight representative metabolites were quantitatively analyzed (Figure 3). Eight selected metabolites were quantified using targeted liquid chromatography–tandem mass spectrometry (LC–MS/MS) with authentic reference standards and an independent set of biological samples (*n* = 5 per group). The corresponding calibration curves are presented in Supplementary Figure S2, and their regression equations, coefficients of determination (R^2^), and calibration ranges are summarized in Supplementary Table S2. Representative multiple reaction monitoring (MRM) chromatograms of the reference standards and sample extracts are provided in Supplementary Figure S3. These chromatograms document the analytical signals used for peak integration and quantification of the selected metabolites.

Of the eight metabolites examined, seven exhibited statistically significant differences between WT and AC samples after false discovery rate (FDR) correction (q < 0.05; Figure 3 and Supplementary Table S2). Among the metabolites showing higher contents in AC samples, adenosine increased from 3.63 ± 1.98 μg/g dry weight (DW) in WT to 15.24 ± 5.02 μg/g DW in AC (q = 0.0070; Figure 3A). Cordycepin was also more abundant in AC (4.93 ± 0.72 μg/g DW) than in WT (3.53 ± 0.53 μg/g DW; q = 0.0121; Figure 3B). Similarly, ergosterol exhibited higher contents in AC (278.84 ± 8.51 μg/g DW) than in WT (118.04 ± 4.76 μg/g DW; q < 0.0001; Figure 3C).

In contrast, four metabolites showed significantly higher contents in WT samples. Hypoxanthine contents were 62.88 ± 33.58 μg/g DW in WT and 16.57 ± 6.11 μg/g DW in AC (q = 0.0406; Figure 3D). Deoxyguanosine was more abundant in WT (29.93 ± 1.97 μg/g DW) than in AC (8.52 ± 2.97 μg/g DW; q < 0.0001; Figure 3F). Likewise, guanosine (304.38 ± 34.42 versus 186.40 ± 18.18 μg/g DW; q = 0.0013; Figure 3G) and guanine (82.20 ± 26.58 versus 17.65 ± 7.06 μg/g DW; q = 0.0070; Figure 3H) were significantly more abundant in WT than in AC samples. Overall, these findings provide quantitative evidence of differences in the abundance of selected metabolites between the analyzed WT and AC *O. sinensis* samples.

### 2.4. Identification of Potential Therapeutic Targets and Key Hub Targets

Based on differential metabolic profiling, targeted quantitative validation, database annotation, and literature-supported biological relevance, eight candidate metabolites associated with liver fibrosis were selected for subsequent pharmacological mechanism analysis. These metabolites included adenosine, uridine diphosphate N-acetylglucosamine (UDP-N-acetylglucosamine), pyroglutamic acid, hypoxanthine, N-acetyl-D-glucosamine, ergosterol, guanosine, and cordycepin (Supplementary Figure S4, Supplementary Tables S2 and S3). These metabolites were subsequently considered as candidate bioactive constituents for pharmacological mechanism analysis.

**Figure 4:**
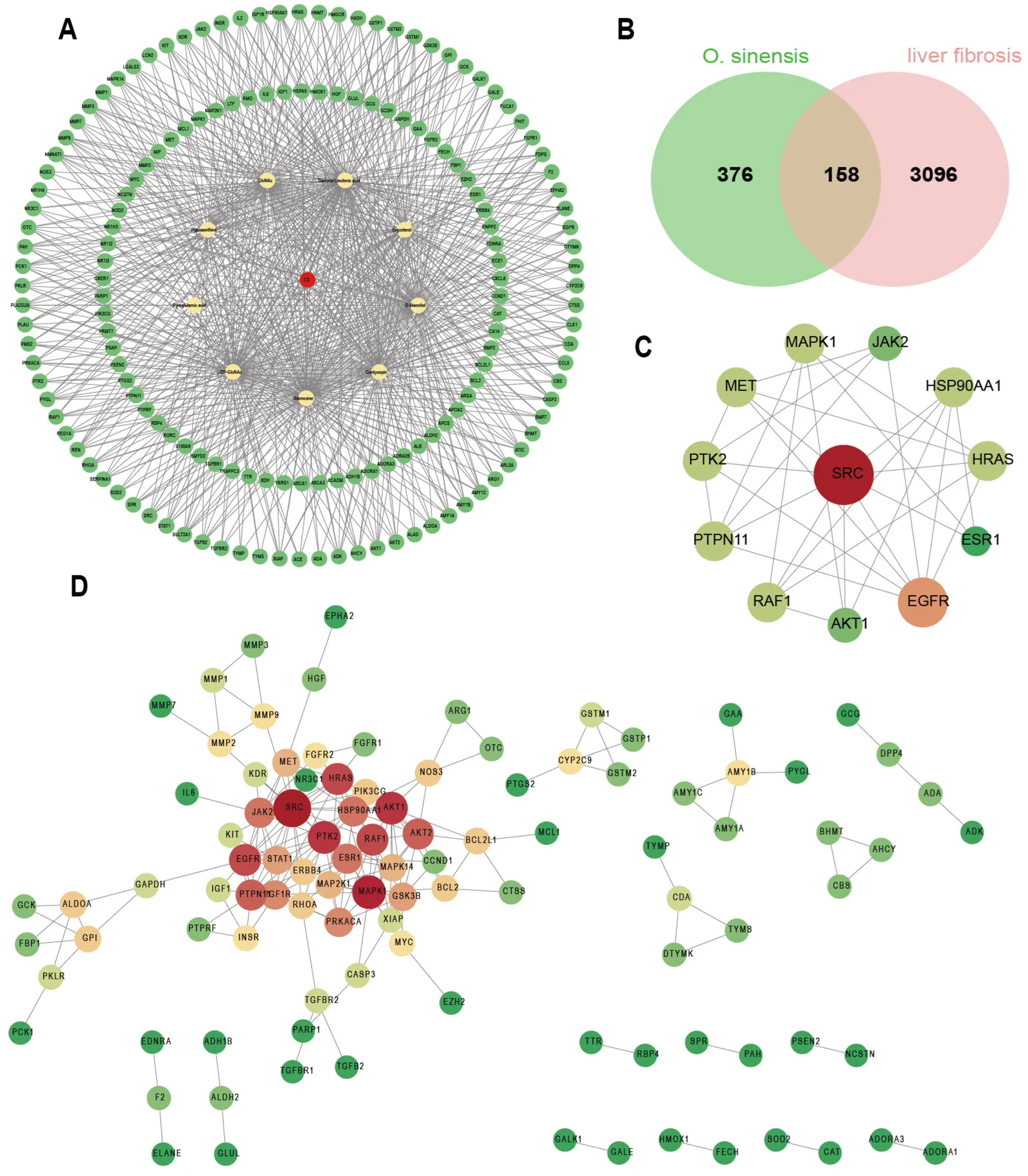
Network pharmacology analysis of the potential targets of core active constituents against liver fibrosis. (**A**) Predicted metabolite-target network. (**B**) Venn diagram between predicted targets of *O. sinensis* core active constituents and liver fibrosis-related targets. (**C**) Hub-node network. (**D**) Protein–protein interaction network of the 158 overlapping targets. In panels A, C, and D, node colors represent degree values, with the color gradient from green through yellow to red indicating progressively higher degree values.

To explore the potential molecular mechanism of the candidate bioactive metabolites of *O. sinensis* in liver fibrosis, target prediction and disease-target intersection analysis were performed. A total of 534 potential compound-related targets were obtained, and 3254 liver fibrosis-related targets were collected from disease databases. Venn analysis identified 158 overlapping targets between the compound-related targets and liver fibrosis-related targets, which were considered candidate therapeutic targets of *O. sinensis* against liver fibrosis (Figure 4B).

Based on these overlapping targets, a metabolite–target interaction network was constructed to visualize the relationships between the candidate bioactive metabolites and their potential liver fibrosis-related targets. The network showed that multiple metabolites were connected with multiple targets, and several targets were shared by different metabolites, suggesting a multi-component and multi-target regulatory pattern of *O. sinensis* (Figure 4A).

To further investigate the interactions among the 158 overlapping targets, a protein–protein interaction (PPI) network was constructed. The PPI network displayed extensive interactions among the candidate targets and contained several highly connected nodes within the central region of the network (Figure 4D). Hub targets were then screened using cytoHubba based on five topological algorithms, including Betweenness, Closeness, Degree, maximal clique centrality (MCC), and maximum neighborhood component (MNC). The top-ranked targets obtained from these algorithms were integrated and re-ranked to identify the most important nodes in the network. Finally, 12 hub targets were selected, including proto-oncogene tyrosine-protein kinase Src (SRC), AKT serine/threonine kinase 1 (AKT1), epidermal growth factor receptor (EGFR), mitogen-activated protein kinase 1 (MAPK1), protein tyrosine kinase 2 (PTK2), HRas proto-oncogene, GTPase (HRAS), HRas proto-oncogene, GTPase (RAF1), protein tyrosine phosphatase non-receptor type 11 (PTPN11), heat shock protein 90 alpha family class A member 1 (HSP90AA1), estrogen receptor 1 (ESR1), Janus kinase 2 (JAK2), and MET proto-oncogene, receptor tyrosine kinase (MET) (Figure 4C; Supplementary Table S4).

### 2.5. Functional Enrichment Identifies Kinase-Centered Pathways Associated with Predicted Targets

**Figure 5:**
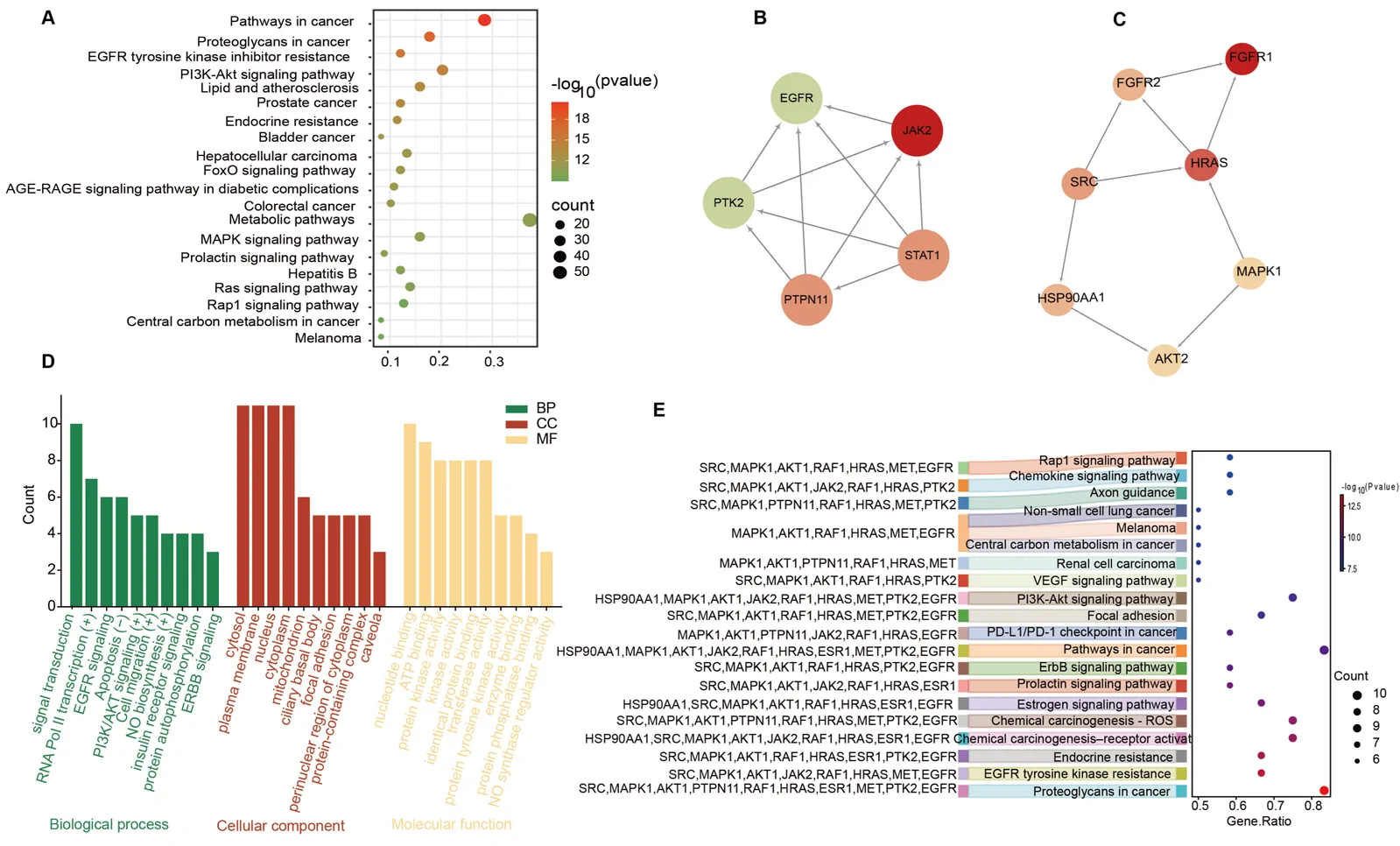
Functional enrichment and network-module analysis of predicted fibrosis-related candidate targets associated with selected *O. sinensis* metabolites. (**A**) KEGG enrichment analysis of the 158 candidate targets. (**B**,**C**) Representative MCODE clusters 1 and 3 derived from the PPI network. node colors represent degree values, with the color gradient from green through yellow to red indicating progressively higher degree values. (**D**) GO enrichment analysis of the 12 hub targets, including biological process (BP), cellular component (CC), and molecular function (MF) categories. (**E**) KEGG enrichment analysis of the 12 hub targets; dot size indicates target number and color represents −log10(*p* value).

To predict the biological functions and potential signaling pathways associated with the candidate targets of *O. sinensis* against liver fibrosis, Gene Ontology (GO) and KEGG enrichment analyses were first performed using the 158 overlapping targets. KEGG enrichment analysis showed that these targets were mainly enriched in pathways associated with receptor tyrosine kinase signaling and downstream intracellular signaling cascades, including pathways in cancer, proteoglycans in cancer, EGFR tyrosine kinase inhibitor resistance, the phosphatidylinositol 3-kinase/protein kinase B (PI3K-Akt) signaling pathway, lipid and atherosclerosis, the forkhead box O (FoxO) signaling pathway, the advanced glycation end products (AGE)–receptor for advanced glycation end products (RAGE) signaling pathway, the mitogen-activated protein kinase (MAPK) signaling pathway, Ras signaling pathway, and Rap1 signaling pathway (Figure 5A). GO enrichment analysis further showed that these targets were mainly involved in signal transduction, transcriptional regulation, protein phosphorylation, kinase-mediated signaling, inflammatory response, cell proliferation, and cell migration. Their molecular functions were primarily associated with protein kinase activity, ATP binding, nucleotide binding, and protein tyrosine kinase activity (Supplementary Figure S5A). These results suggest that the 158 overlapping targets are functionally concentrated in phosphorylation-dependent signal transduction and kinase-related regulatory processes.

Next, functional enrichment analysis was performed for the 12 cytoHubba-defined hub targets, including SRC, AKT1, EGFR, MAPK1, PTK2, HRAS, RAF1, PTPN11, HSP90AA1, ESR1, JAK2, and MET. GO enrichment analysis showed that these hub targets were mainly associated with signal transduction, the EGFR signaling pathway, the MAPK cascade, protein autophosphorylation, and transcriptional regulation. Their enriched molecular functions included nucleotide binding, protein kinase binding, protein kinase activity, protein tyrosine kinase activity, and protein phosphatase binding (Figure 5D). KEGG enrichment analysis further indicated that the hub targets were enriched in the Rap1 signaling pathway, chemokine signaling pathway, vascular endothelial growth factor (VEGF) signaling pathway, PI3K-Akt signaling pathway, focal adhesion, ErbB signaling pathway, EGFR tyrosine kinase inhibitor resistance, endocrine resistance, chemical carcinogenesis-reactive oxygen species, and proteoglycans in cancer (Figure 5E). These results indicate that the core targets were closely associated with receptor tyrosine kinase-related signaling, PI3K-Akt/MAPK/Ras cascades, inflammatory regulation, oxidative stress, cell adhesion, and migration-related processes.

To further validate the functional consistency of the target network, Molecular Complex Detection (MCODE) analysis was performed to identify highly connected modules within the PPI network. Two representative clusters were obtained: Cluster 1 contained EGFR, PTK2, JAK2, signal transducer and activator of transcription 1 (STAT1), and PTPN11, whereas Cluster 3 contained fibroblast growth factor receptor 1 (FGFR1), fibroblast growth factor receptor 2 (FGFR2), SRC, HRAS, HSP90AA1, MAPK1, and AKT serine/threonine kinase 2 (AKT2) (Figure 5B,C; Supplementary Table S5). Functional enrichment analysis of these two MCODE-derived clusters showed that they were mainly enriched in receptor tyrosine kinase signaling, Janus kinase–signal transducer and activator of transcription (JAK–STAT) signaling pathway, PI3K-Akt signaling pathway, MAPK cascade, Ras/Rap1 signaling, VEGF signaling, and kinase activity-related terms (Supplementary Figure S5B–D). These module-level enrichment results were consistent with the findings obtained from the 158 overlapping targets and 12 hub targets, further highlighting the predicted association of growth factor receptor-related kinase signaling networks with the potential mechanisms underlying the antifibrotic properties of *O. sinensis*.

### 2.6. Molecular Docking and Molecular Dynamics Simulations

Molecular docking was performed to evaluate the predicted interactions between the candidate bioactive constituents and six hub targets. In total, 34 ligand–target pairs were successfully docked, with Vina docking scores ranging from −4.1 to −10.3 kcal/mol (Supplementary Table S6 and Supplementary Figure S6). Thirteen complexes yielded docking scores of ≤−7.0 kcal/mol, placing them among the more favorable predicted interactions within the present docking dataset. UDP-N-acetylglucosamine exhibited the most favorable overall docking profile, particularly with HRAS (−10.3 kcal/mol), AKT1 (−9.3 kcal/mol), and EGFR (−8.3 kcal/mol). Ergosterol also showed favorable docking scores with HRAS (−8.9 kcal/mol), SRC (−8.8 kcal/mol), and AKT1 (−7.9 kcal/mol). At the target level, HRAS, AKT1, and SRC were among the proteins showing the most favorable overall docking profiles. Representative docking poses suggested that these ligands could be accommodated within the corresponding binding pockets and establish contacts with surrounding residues (Supplementary Figure S6). Based on docking score, target relevance within the predicted signaling network, and ligand diversity, three representative complexes: HRAS-UDP-N-acetylglucosamine, AKT1–UDP-N-acetylglucosamine, and SRC–ergosterol were selected for subsequent MD simulations.

**Figure 6:**
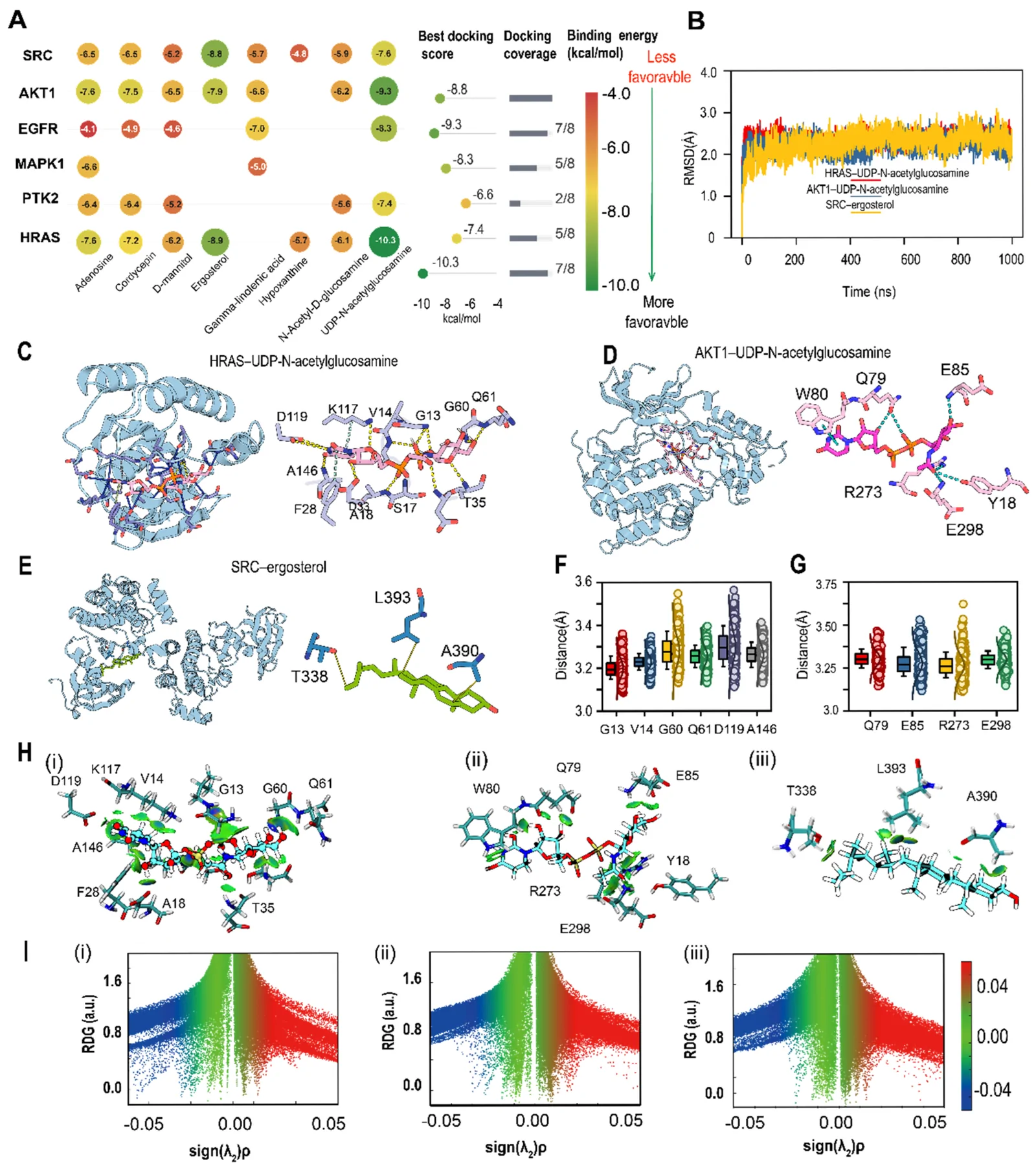
Molecular dynamics-based assessment of representative metabolite-target complexes. (**A**) Bubble plot of molecular docking scores for representative active constituents against six hub targets, including SRC, AKT1, EGFR, MAPK1, PTK2, and HRAS. More negative docking scores indicate more favorable predicted binding. (**B**) Protein-backbone RMSD profiles obtained from three independent MD simulation replicas for the HRAS–UDP-N-acetylglucosamine, AKT1–UDP-N-acetylglucosamine, and SRC–ergosterol complexes. (**C**–**E**) Representative conformations extracted by cluster analysis of the MD trajectories for (**C**) HRAS–UDP-N-acetylglucosamine, (**D**) AKT1–UDP-N-acetylglucosamine, and (**E**) SRC–ergosterol. Key ligand–residue contacts are indicated by dashed lines. (**F**,**G**) Time-dependent distance distributions of representative intermolecular contacts in the (**F**) HRAS–UDP-N-acetylglucosamine and (**G**) AKT1–UDP-N-acetylglucosamine complexes during the MD simulations. (**H**) Independent gradient model based on Hirshfeld partitioning (IGMH) analysis of noncovalent interactions in the (**I**). The RDG plot of (i) HRAS–UDP-N-acetylglucosamine, (ii) AKT1–UDP-N-acetylglucosamine, and (iii) SRC–ergosterol complexes. The sign(λ_2_)ρ(r)-mapped δg_inter isosurfaces illustrate the spatial distribution and nature of intermolecular interactions. Blue and green regions predominantly represent attractive interactions, whereas red regions indicate steric repulsion. The corresponding scatter plots further illustrate the distribution and relative characteristics of the noncovalent interactions.

To examine the dynamic behavior of the selected docking poses under explicit-solvent conditions, multi-replica MD simulations were performed for the HRAS–UDP-N-acetylglucosamine, AKT1–UDP-N-acetylglucosamine, and SRC–ergosterol complexes. These systems were selected to represent two high-scoring UDP-N-acetylglucosamine complexes involving distinct signaling nodes, together with a chemically distinct sterol ligand interacting with the SRC kinase. Across the three independent replicas of each system, protein-backbone root-mean-square deviation (RMSD) profiles reached relatively stable plateaus after approximately 25 ns (Figure 6B). No sustained upward drift was observed during the remaining simulation period, indicating that the three complexes sampled comparatively stable protein conformational ensembles. Consistently, the radius of gyration (Rg) remained largely stable, with no sustained expansion or collapse of the protein structures, further supporting the overall conformational stability of the simulated systems (Supplementary Figure S7).

Representative conformations were subsequently extracted by cluster analysis to characterize the predominant protein–ligand interaction patterns sampled during the MD trajectories. The resulting structures revealed distinct, system-specific networks of noncovalent contacts between each ligand and residues within the surrounding binding pocket. In the HRAS–UDP-N-acetylglucosamine complex, an extensive hydrogen-bonding network was observed around the phosphate-containing region of the ligand. The backbone nitrogen atoms of Val14, Gly15, and Lys16 formed N–H···O hydrogen bonds with phosphate oxygen atoms, whereas the backbone nitrogen atoms of Ala145 and Lys146 interacted with oxygen atoms located at the distal region of UDP-N-acetylglucosamine. The corresponding heavy-atom distances were predominantly distributed within approximately 3.3–3.6 Å during the simulations, indicating frequent sampling of these contacts (Figure 6F).

In the AKT1–UDP-N-acetylglucosamine complex, the aromatic side chain of Trp80 adopted a near-parallel orientation relative to the ligand, consistent with a stacking-like aromatic interaction. The OE1 atom of Gln79 participated in a bifurcated hydrogen-bonding arrangement involving two oxygen-containing groups of UDP-N-acetylglucosamine. Additional hydrogen-bonding contacts involved the guanidinium group of Arg273, the backbone nitrogen atom of Glu298, and the phenolic oxygen atom of Tyr18. Heavy-atom distance analysis showed that these contacts were frequently maintained within geometries compatible with hydrogen bonding throughout the MD trajectories (Figure 6G).

In contrast, the SRC–ergosterol complex exhibited a simpler interaction pattern dominated by close hydrophobic and van der Waals contacts. Ergosterol remained positioned near Thr338, Ala390, and Leu393, with representative contacts involving the OG1 atom of Thr338, the CB atom of Ala390, and the CD2 atom of Leu393. Collectively, these interactions contributed to the retention of ergosterol within the SRC binding pocket during the simulations.

To further characterize the nature and spatial distribution of noncovalent interactions in the representative complexes, an independent gradient model based on Hirshfeld partitioning (IGMH) analysis was performed [23]. The HRAS–UDP-N-acetylglucosamine complex exhibited the most spatially extensive intermolecular interaction region, followed by the AKT1–UDP-N-acetylglucosamine complex, whereas the SRC–ergosterol complex displayed a more localized interaction region (Figure 6H). These differences were consistent with the distinct physicochemical properties and binding modes of UDP-N-acetylglucosamine and ergosterol. Within the IGMH framework, the sign(λ_2_)ρ(r)-mapped δg_inter isosurfaces provide information on both the spatial distribution and character of intermolecular interactions [23]. Blue-to-green regions at the ligand–residue interfaces were predominantly associated with attractive noncovalent interactions, including hydrogen bonding and van der Waals contacts, whereas regions with positive sign(λ_2_)ρ(r) values were associated with repulsive or sterically unfavorable interactions. The IGMH isosurfaces and corresponding scatter plots therefore complemented the trajectory-based analyses by providing a real-space visualization of the distribution and character of intermolecular interactions in the three representative complexes.

At the ligand level, UDP-N-acetylglucosamine displayed the most favorable and broad binding profile, interacting strongly with HRAS, AKT1, EGFR, SRC, and PTK2. Ergosterol also showed strong binding affinities with HRAS, SRC, and AKT1. In contrast, D-mannitol, hypoxanthine, and N-acetyl-D-glucosamine generally showed relatively weaker binding affinities in the current docking analysis. At the target level, HRAS and AKT1 showed the most favorable overall docking profiles, followed by SRC and PTK2, indicating that these proteins may represent important binding targets for the active constituents of *O. sinensis*. Representative docking conformations further showed that the selected ligands could be accommodated within the binding pockets of their corresponding targets and formed multiple interactions with surrounding amino acid residues (Figure 6B–F and Supplementary Figure S7). These results support the network pharmacology findings and suggest that the core active constituents of *O. sinensis*, particularly UDP-N-acetylglucosamine and ergosterol, may interact with key targets such as HRAS, AKT1, SRC, EGFR, and PTK2, providing a structural basis for their potential regulatory effects on fibrosis-related signaling pathways.

## 3. Discussion

Previous studies have demonstrated substantial metabolic differences between wild and cultivated *O. sinensis*, including differences in differential metabolites, volatile components, and major classes of bioactive constituents [24,25]. Consistent with these observations, the present study identified 2193 differential metabolites and revealed marked source-dependent metabolic differences between wild and cultivated *O. sinensis*. Importantly, targeted quantitative analysis further confirmed differences in several representative metabolites. Adenosine, cordycepin, and ergosterol were significantly more abundant in AC, whereas hypoxanthine was enriched in WT; pyroglutamic acid also showed a higher level in AC, although the difference was not statistically significant. The quantitative trends of deoxyguanosine, guanosine, and guanine were also consistent with the untargeted metabolomic profiles. These results indicate that wild and cultivated *O. sinensis* differ not only in global metabolic composition but also in the abundance of pharmacologically relevant metabolites.

The pharmacological significance of these compositional differences is particularly relevant to liver fibrosis. *O. sinensis* has long been used in the management of liver-related disorders, and experimental studies have reported hepatoprotective and anti-fibrotic effects of *O. sinensis*-derived polysaccharides and cultured mycelial preparations [15,16,26]. Several characteristic constituents have also been implicated in fibrosis-related processes. For example, cordycepin has been reported to attenuate hepatic steatosis, inflammation, and fibrosis through adenosine monophosphate-activated protein kinase (AMPK)-related signaling, inhibition of glutaminolysis, and induction of hepatic stellate cell senescence, as well as modulation of the SOX9/Wnt/β-catenin axis [27,28,29]. Nevertheless, previous comparisons of wild and cultivated *O. sinensis* have mainly focused on chemical composition [24,25], and whether source-dependent metabolite differences translate into different anti-fibrotic molecular mechanisms has remained unclear.

Network pharmacology and functional enrichment analyses in the present study suggested that the candidate bioactive metabolites may regulate liver fibrosis through a multi-component and multi-target mechanism. Liver fibrosis is characterized by persistent inflammation, hepatic stellate cell activation, excessive extracellular matrix deposition, and tissue remodeling [30,31]. Consistent with these pathological processes, the overlapping targets were enriched in PI3K-Akt, MAPK, Ras, Rap1, ErbB, VEGF, focal adhesion, chemokine, and JAK-STAT signaling pathways (Figure 5A). Among these pathways, PI3K-Akt and MAPK signaling are particularly relevant to growth factor responses, cell survival, proliferation, migration, and fibrogenic activation [32,33]. Growth factor receptor-associated signaling can activate downstream PI3K-Akt and MAPK cascades and thereby promote phosphorylation-dependent responses involved in hepatic stellate cell activation and matrix remodeling [34]. The consistency among enrichment analyses of the overlapping targets, hub targets, and MCODE modules further suggests that the predicted effects of *O. sinensis* are more likely mediated through coordinated regulation of interconnected kinase-centered signaling networks rather than isolated molecular targets.

Molecular docking provided a structure-based assessment of the feasibility of several network-predicted metabolite–target interactions. Several candidate metabolites, particularly UDP-N-acetylglucosamine and ergosterol, exhibited favorable docking scores with HRAS, AKT1, SRC, EGFR, and PTK2. These proteins occupy functionally interconnected positions within growth factor receptor- and kinase-associated signaling networks: EGFR, SRC, and PTK2 participate in upstream receptor tyrosine kinase and focal adhesion-associated signaling [35,36]; HRAS links upstream signals to activation of the Ras/MAPK cascade; and AKT1 is a major downstream effector of PI3K-Akt signaling [37]. Multi-replica MD simulations of the representative HRAS–UDP-N-acetylglucosamine, AKT1–UDP-N-acetylglucosamine, and SRC–ergosterol complexes further showed that the selected docking-derived conformations could be maintained within comparatively stable protein conformational ensembles and were supported by persistent, system-specific noncovalent interactions. Together, the network pharmacology, molecular docking, and MD simulation results support the EGFR/SRC/PTK2–HRAS–PI3K-Akt/MAPK signaling axis as a hypothesis-generating mechanistic framework linking candidate metabolites of *O. sinensis* to liver fibrosis-related processes.

Based on the above mechanistic framework of liver fibrosis, we further analyzed the differences between wild-collected and cultivated *O. sinensis*. Several metabolites enriched in cultivated *O. sinensis* were also among those showing favorable interactions with key fibrosis-related targets. Ergosterol was predicted to interact with HRAS, SRC, and AKT1, whereas adenosine and cordycepin showed favorable interactions with AKT1 and HRAS. UDP-N-acetylglucosamine, which was enriched in AC according to untargeted metabolomics, also displayed strong predicted interactions with HRAS, AKT1, EGFR, SRC, and PTK2. These findings suggest that the metabolite composition of cultivated *O. sinensis* may be more closely associated with regulation of the EGFR/SRC/PTK2–HRAS–PI3K-Akt/MAPK signaling network. In contrast, wild *O. sinensis* was characterized by higher levels of hypoxanthine and several other metabolites, including γ-linolenic acid, N-acetyl-D-glucosamine, D-mannitol, deoxyguanosine, guanosine, and guanine. Among them, γ-linolenic acid showed a favorable predicted interaction with EGFR. Thus, WT and AC may differ not only in the abundance of specific metabolites but also in the potential biological mechanisms associated with their major bioactive constituents.

WT and AC exhibit distinct metabolic profiles. Network pharmacology and molecular dynamics (MD) analyses focusing on liver fibrosis provide clearer insights into their divergent therapeutic potential against liver fibrosis. Cultivated *O. sinensis* showed a more prominent predicted association with kinase-centered signaling, particularly the EGFR/SRC/PTK2–HRAS–PI3K-Akt/MAPK axis, whereas wild *O. sinensis* exhibited a distinct metabolic profile that may involve additional metabolic or inflammatory regulatory mechanisms. WT and AC exhibit distinct metabolic profiles. Integrated network pharmacology and MD analyses focusing on liver fibrosis provided insights into the potential biological relevance of these source-associated metabolites. Cultivated *O. sinensis* showed a more prominent predicted association with kinase-centered signaling, particularly the EGFR/SRC/PTK2–HRAS–PI3K-Akt/MAPK axis, whereas wild *O. sinensis* exhibited a distinct metabolic profile that may involve additional metabolic or inflammatory regulatory mechanisms. However, these predicted mechanistic differences should not be interpreted as direct evidence that cultivated *O. sinensis* possesses superior anti-fibrotic efficacy. Moreover, the artificially cultivated *O. sinensis* samples analyzed in this study were obtained from commercial sources rather than directly cultivated by our research group. Therefore, variations associated with cultivation conditions, production batches, and sample provenance may potentially contribute to the observed metabolic differences. Further comparative validation using independently characterized samples and appropriate cellular and animal models of liver fibrosis will be required to determine their biological significance. Furthermore, UDP-N-acetylglucosamine was included solely for exploratory pathway analysis. As a highly polar, negatively charged nucleotide sugar, it cannot readily cross cell membranes via passive diffusion and was therefore not considered a candidate with established cellular accessibility or direct antifibrotic activity.

## 4. Materials and Methods

### 4.1. Chemicals, Reagents, and Instruments

Methanol (chromatographic grade; Chemical Abstracts Service (CAS) registry no.: 67-56-1; Fisher Scientific, Waltham, MA, USA; catalog no. A456-4), acetonitrile (chromatographic grade; CAS: 75-05-8; Fisher Scientific; catalog no. A955-4), formic acid (mass spectrometry grade; CAS: 64-18-6; Tokyo Chemical Industry Co., Ltd., Tokyo, Japan; catalog no. F0654), and the internal standard L-2-chlorophenylalanine (purity 98.0%; CAS: 103616-89-3; Aladdin Biochemical Technology Co., Ltd., Shanghai, China; catalog no. C105993) were used. All other reagents were of analytical grade.

Authentic reference standards of cordycepin (3′-deoxyadenosine; CAS No. 73-03-0; 99.5% purity by HPLC), ergosterol (CAS No. 57-87-4; analytical standard), and L-pyroglutamic acid (5-oxoproline; CAS No. 98-79-3; ≥99% purity) were purchased from Shanghai Macklin Biochemical Technology Co., Ltd. (Shanghai, China). Adenosine (CAS No. 58-61-7; ≥99% purity) and hypoxanthine (CAS No. 68-94-0; ≥99% purity) were obtained from Shanghai Aladdin Biochemical Technology Co., Ltd. (Moligand™ brand; Shanghai, China). Guanosine (CAS No. 118-00-3; 99.4% purity by HPLC) and guanine (CAS No. 73-40-5; 99.35% purity by HPLC) were obtained from Weiye Metrology (Beijing, China). 2′-Deoxyguanosine (CAS No. 961-07-9; 99.90% purity) was purchased from MedChemExpress LLC (Monmouth Junction, NJ, USA).

### 4.2. Sample Collection and Pretreatment

Sample collection and pretreatment were conducted following the methods described in our previous publication. Samples were collected during the early fruiting stages when the stroma had just begun to produce spores [7,38]. Specifically, WT samples were collected in June 2025 from Jiali County, Nagqu city, Xizang Autonomous Region. A minimum of 20 WT samples were collected from three randomly selected plots, each separated by 100 m. Within each plot, 3–6 samples were collected in a random manner. The collected samples were preserved in ice chests with sterile plastic bags and promptly transported to the Plant Biotechnology R&D Center laboratory at Shanghai Jiao Tong University. After removing the external mycelial cortices along with the attached soil, the intact specimens were oven-dried at 45 °C for 48 h, ground into a fine powder using a tissue grinder, and stored at −20 °C until use. AC samples were procured from various pharmacies in the medicinal materials market of Lhasa City, Tibet Autonomous Region, in the same year. Six biological replicates per group were used for untargeted metabolomics, and each biological replicate comprised material from one individual specimen.

### 4.3. Untargeted Metabolomics Analysis by UHPLC-Orbitrap HRMS

About 10 mg of each evenly pulverized, oven-dried powder sample (WT and AC *O. sinensis*) was precisely measured and placed into a 2 mL centrifuge tube [39]. Each tube received 300 μL of a chilled extraction solvent (methanol/acetonitrile/water, 2:2:1, *v*/*v*/*v*) infused with 5 ppm L-2-chlorophenylalanine (internal standard) along with two stainless steel grinding beads. The blend was vortexed for 30 s and subsequently homogenized with a high-throughput tissue grinder at 55 Hz for 60 s; the homogenization process was conducted a second time. The tubes were then exposed to ultrasonication for 10 min, after which they were incubated at −20 °C for 30 min to facilitate the precipitation of proteins and other contaminants. Following incubation, the samples underwent centrifugation at 12,000 rpm and 4 °C for a duration of 10 min. The supernatant was gathered, subjected to filtration using a 0.22 μm membrane, and thereafter placed into a sterile vial for examination. In batches including six samples, a quality control (QC) specimen was created by amalgamating 10–20 μL of filtrate from every sample. The QC sample was employed to assess instrument stability and data dependability during the analytical process.

Chromatographic separation was performed on an ACQUITY UPLC HSS T3 column (100 Å, 1.8 µm, 2.1 mm × 100 mm; Waters, Milford, MA, USA). Mass spectrometric analysis was conducted on a Thermo Orbitrap Exploris 120 instrument (Thermo Fisher Scientific) equipped with a heated electrospray ionization (HESI) source and controlled by Xcalibur software (version 4.7) [40]. Data were acquired in data-dependent acquisition (DDA) mode, alternating between positive and negative ion modes. Raw data experienced peak extraction, alignment, filtering, gap filling, normalization, and metabolite annotation with reference to the PerSonalbio Next-Generation Metabolomics Database (PSNGM).

### 4.4. Targeted Quantification by UPLC-MS/MS

Eight metabolites, including adenosine, cordycepin, ergosterol, hypoxanthine, pyroglutamic acid, deoxyguanosine, guanosine, and guanine, were quantified using external calibration curves generated from serially diluted authentic standards. Targeted LC–MS/MS analysis was performed on an ACQUITY UPLC Premier system coupled to a Xevo TQ-Absolute triple-quadrupole mass spectrometer (Waters Corporation, Milford, MA, USA), with chromatographic separation achieved on an ACQUITY UPLC BEH C18 column (2.1 × 100 mm, 1.7 μm, Waters Corporation, Milford, MA, USA). Data were acquired in positive-ion multiple reaction monitoring mode and processed using MassLynx 4.2 software. Detailed calibration procedures, chromatographic conditions, mass spectrometric parameters, and optimized MRM transitions are provided in Supplementary Method S1. Targeted quantification was performed using a new, independent sample set with five biological replicates per group; each biological replicate comprised material from one individual specimen.

### 4.5. Screening of Compound-Related Targets and Disease-Related Targets

Active candidates were screened from differential metabolites using adjusted *p* < 0.05, |log_2_ fold change (FC)| ≥ 1, variable importance in projection (VIP) > 1, and the availability of both Human Metabolome Database (HMDB) and KEGG identifiers. SwissADME [41] was then used to evaluate the physicochemical properties, predicted gastrointestinal absorption, and drug-likeness of the candidate compounds, including their compliance with Lipinski’s rule of five. In parallel, a literature-based assessment was conducted to evaluate their reported pharmacological activities and biological relevance to liver fibrosis. Metabolites with unfavorable predicted oral absorption properties could be retained for exploratory molecular analysis when supported by metabolomic evidence and relevant biological literature.

### 4.6. Collection of Compound-Related Targets and Disease-Related Targets

Potential targets of the identified core active constituents were predicted using SwissTargetPrediction [42] (https://www.swisstargetprediction.ch/, accessed on 11 September 2025), PharmMapper [43] (https://www.lilab-ecust.cn/pharmmapper/, accessed on 13 September 2025), and the Drug–Gene Interaction Database DGIdb [44] (https://dgidb.org/, accessed on 14 September 2025). The predicted targets obtained from different databases were merged, and duplicate entries were removed to generate the potential target set of the core active constituents.

Liver fibrosis-related targets were collected from the Online Mendelian Inheritance in Man (OMIM [45], https://www.omim.org/, accessed on 14 September 2025) and GeneCards [46] databases (https://www.genecards.org/, accessed on 14 September 2025) using “liver fibrosis” as the search keyword. Targets with a relevance score greater than 0 in the GeneCards database were extracted and integrated with the targets obtained from OMIM. After removing duplicates, a liver fibrosis-related target set was generated.

### 4.7. Screening of Overlapping Targets and Network Construction

To identify potential targets through which the core active constituents of *O. sinensis* may act against liver fibrosis, the predicted targets of the core active constituents were intersected with liver fibrosis-related targets using Venn analysis [47]. The overlapping targets were considered candidate targets of the core active constituents against liver fibrosis and were used for subsequent network pharmacology and molecular docking analyses.

The protein–protein interaction (PPI) network of the overlapping targets was constructed using the Search Tool for the Retrieval of Interacting Genes/Proteins (STRING) database [48] (https://string-db.org/, accessed on 14 September 2025). The species was set to *Homo sapiens*. To ensure the reliability of protein interactions, the minimum required interaction score was set to >0.9. The network type was set as a full STRING network, and the meaning of network edges was set as confidence.

### 4.8. Network Topology Analysis and Hub Target Screening

Cytoscape 3.10.1 [49] was used to construct and visualize the core constituent–target interaction network. A multi-algorithm topology analysis strategy was applied to identify hub nodes. First, the cytoHubba plugin was used to rank candidate nodes according to MCC, MNC, degree centrality, betweenness centrality, and closeness centrality. The top 30 nodes from each ranking were retained, and their intersection was used to define consensus hub targets.

In addition, the MCODE plugin was used to identify densely connected functional modules. The parameters were set as follows: degree cutoff = 2, node score cutoff = 0.2, haircut = true, fluff = false, K-core = 2, and maximum depth from seed = 100.

### 4.9. Functional Enrichment Analysis

Functional enrichment analysis was performed using the Database for Annotation, Visualization and Integrated Discovery Database (DAVID, https://davidbioinformatics.nih.gov/, accessed on 14 September 2025) [50]. Gene Ontology (GO) enrichment analysis, including biological process (BP), cellular component (CC), and molecular function (MF), and KEGG pathway enrichment analysis were conducted for the overlapping targets and subsequently identified hub targets. Significantly enriched terms were selected using a threshold of *p* < 0.05 after Benjamini–Hochberg multiple testing correction. The significantly enriched GO terms and KEGG pathways were ranked in ascending order according to the *p*-value, and the top 20 terms or pathways were selected for visualization and analysis.

### 4.10. Molecular Docking

Molecular docking between candidate bioactive constituents of *O. sinensis* and AKT1 (Protein Data Bank (PDB) ID: 3O96), HRAS (PDB ID: 5P21), EGFR (PDB ID: 1XKK), MAPK1 (PDB ID: 1TVO), PTK2 (PDB ID: 4I4E), and SRC (PDB ID: 4MXO) was performed using AutoDock Vina version 1.2.0 with the Vina scoring function [51,52]. Three representative protein–ligand complexes were selected for subsequent molecular dynamics (MD) simulations [23,53,54,55,56,57] on the basis of their docking scores, target relevance within the predicted signaling network, and ligand diversity: HRAS–UDP-N-acetylglucosamine, AKT1–UDP-N-acetylglucosamine, and SRC–ergosterol. For specific parameter settings, please refer to Supplementary Method S1.

### 4.11. Statistical Analysis

The independent experimental unit was one individual *O. sinensis* specimen. Untargeted metabolomics included six biological replicates per group. For the targeted LC–MS/MS quantitative analysis, five independent biological replicates were included in each group (*n* = 5). Statistical analyses were performed using GraphPad Prism software (version 10.1.2; GraphPad Software, Boston, MA, USA). Differences in metabolite contents between WT and AC *O. sinensis* were evaluated using two-tailed unpaired Welch’s *t*-tests without assuming equal variances. To account for multiple testing across the eight selected metabolites, the resulting *p* values were adjusted using the Benjamini–Hochberg false discovery rate (FDR) procedure. An FDR-adjusted q value below 0.05 was considered statistically significant. Data are presented as mean ± standard deviation (SD).

## 5. Conclusions

This study revealed clear metabolic differences between AC and WT *O. sinensis*, which were further supported by targeted quantification of selected metabolites. Integrated network pharmacology, functional enrichment, molecular docking, and MD simulations further indicated that the distinct metabolite profiles of AC and WT *O. sinensis* may correspond to different target associations and mechanistic characteristics. Metabolites enriched in AC *O. sinensis* showed stronger predicted associations with kinase-centered signaling networks, particularly the EGFR/SRC/PTK2–HRAS–PI3K-Akt/MAPK axis, whereas WT *O. sinensis* exhibited a distinct metabolite-associated network pattern. These findings suggest that source-dependent metabolic variations may contribute to divergent biological characteristics between wild-collected and cultivated *O. sinensis*, providing a basis for understanding their potential functional differences.

## Data Availability

The data supporting the findings of this study are provided within the article and its Supporting Information. The data presented in this study are available from the corresponding author upon reasonable request.

## Supplementary Materials

The following supporting information can be downloaded at: https://www.mdpi.com/article/10.3390/ijms27219558/s1.
