# Supplementary material for: Metabolomic Differences Between Wild and Cultivated Ophiocordyceps sinensis Identify Source-Associated Metabolites with Predicted Antifibrotic Relevance
Source: Int J Mol Sci. 2026 Oct 26;27(21):9558. doi: 10.3390/ijms27219558 (PMC13763523; doi:10.3390/ijms27219558)
Supplement: Supplementary file 1 [file ijms-27-09558-s001.zip › Supplementary Figure S1.pdf]

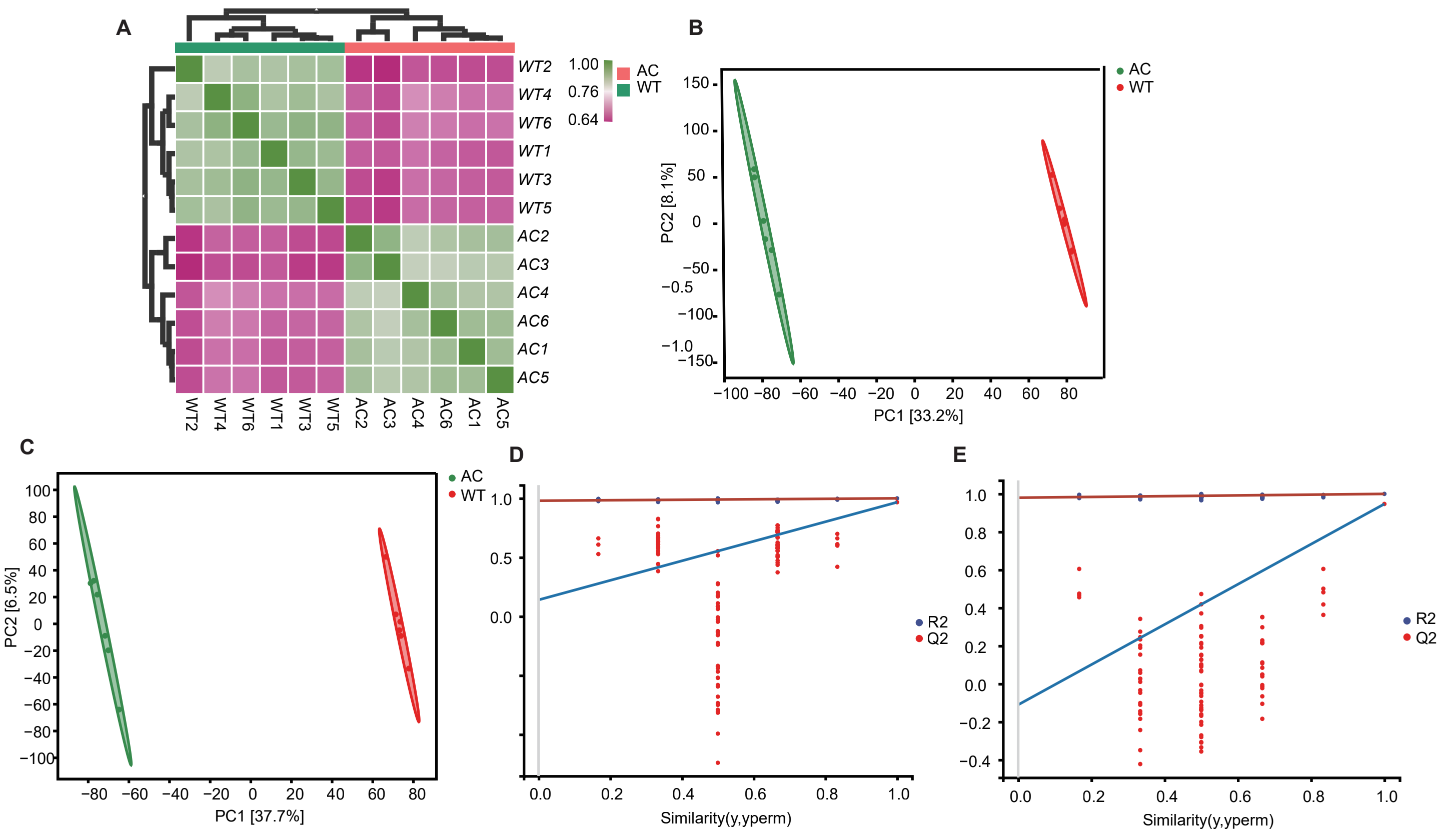
A
WT2
WT4
WT6
WT1
WT3
WT5
AC2
AC3
AC4
AC6
AC1
AC5
1.00
0.76
0.64
AC
WT
B
AC
WT
PC2 [8.1%]
PC1 [33.2%]
C
AC
WT
PC2 [6.5%]
PC1 [37.7%]
D
R2
Q2
Similarity(y,yperm)
E
R2
Q2
Similarity(y,yperm)
