# Supplementary material for: Metabolomic Differences Between Wild and Cultivated Ophiocordyceps sinensis Identify Source-Associated Metabolites with Predicted Antifibrotic Relevance
Source: Int J Mol Sci. 2026 Oct 26;27(21):9558. doi: 10.3390/ijms27219558 (PMC13763523; doi:10.3390/ijms27219558)
Supplement: Supplementary file 1 [file ijms-27-09558-s001.zip › Supplementary Figure S2.pdf]

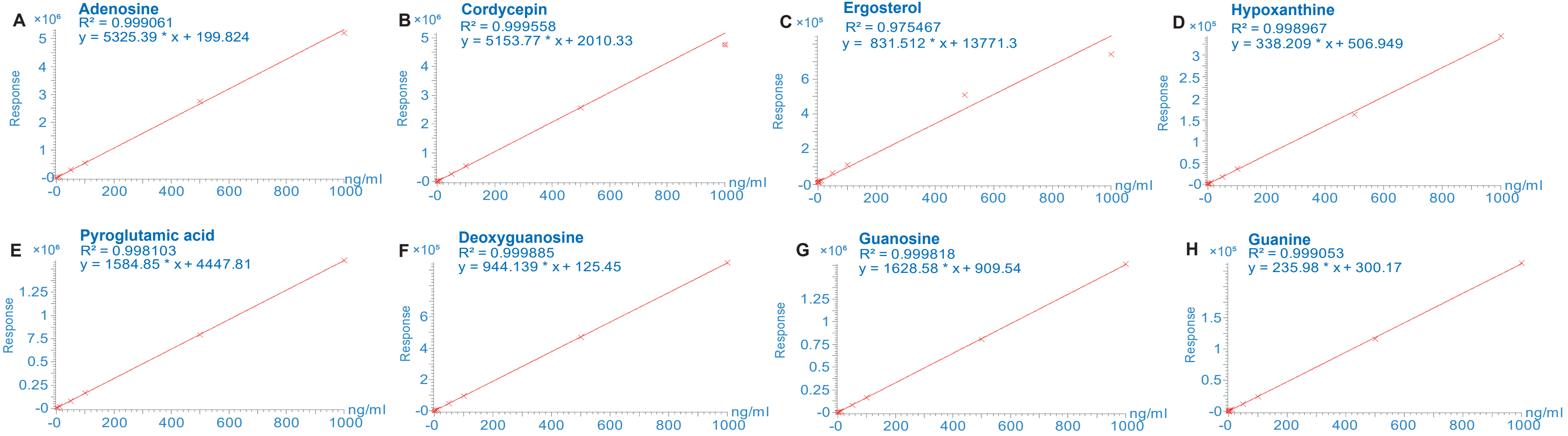

A
Adenosine
R² = 0.999061
y = 5325.39 * x + 199.824
B
Cordycepin
R² = 0.999558
y = 5153.77 * x + 2010.33
C
Ergosterol
R² = 0.975467
y = 831.512 * x + 13771.3
D
Hypoxanthine
R² = 0.998967
y = 338.209 * x + 506.949
E
Pyroglutamic acid
R² = 0.998103
y = 1584.85 * x + 4447.81
F
Deoxyguanosine
R² = 0.999885
y = 944.139 * x + 125.45
G
Guanosine
R² = 0.999818
y = 1628.58 * x + 909.54
H
Guanine
R² = 0.999053
y = 235.98 * x + 300.17
Response
ng/ml
