# Supplementary material for: Metabolomic Differences Between Wild and Cultivated Ophiocordyceps sinensis Identify Source-Associated Metabolites with Predicted Antifibrotic Relevance
Source: Int J Mol Sci. 2026 Oct 26;27(21):9558. doi: 10.3390/ijms27219558 (PMC13763523; doi:10.3390/ijms27219558)
Supplement: Supplementary file 1 [file ijms-27-09558-s001.zip › Supplementary Figure S3.pdf]

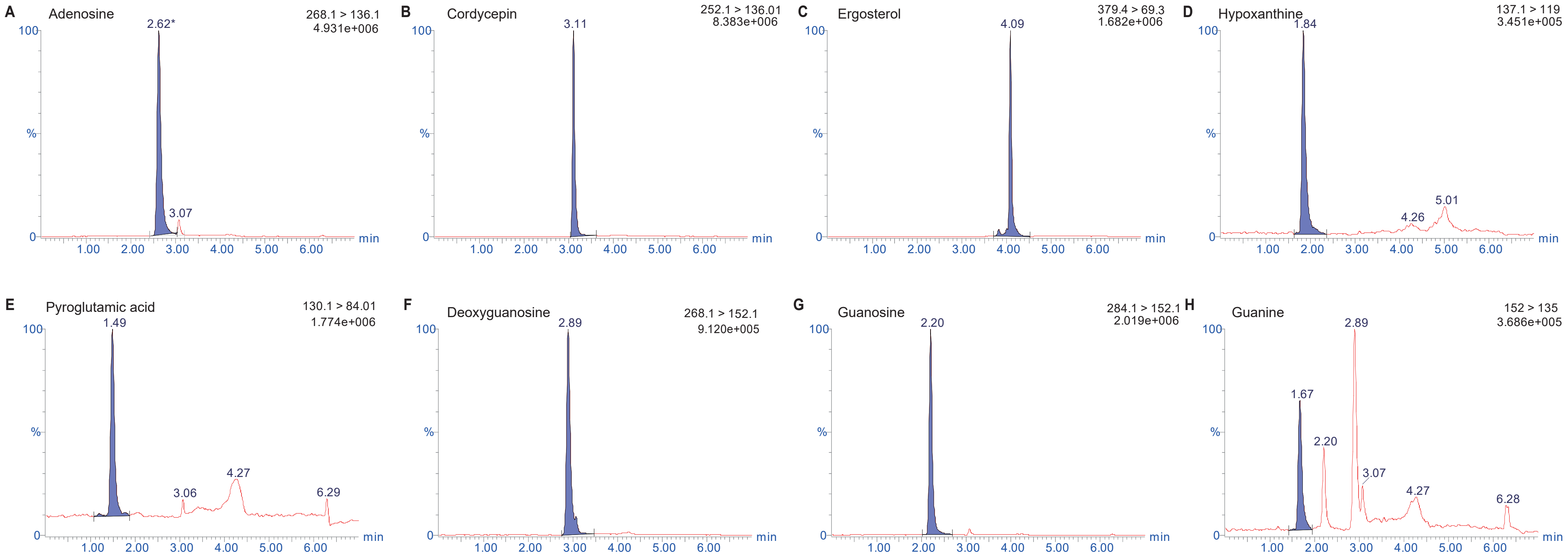
A
Adenosine
268.1 > 136.1
4.931e+006
2.62*
3.07
B
Cordycepin
252.1 > 136.01
8.383e+006
3.11
C
Ergosterol
379.4 > 69.3
1.682e+006
4.09
D
Hypoxanthine
137.1 > 119
3.451e+005
1.84
4.26
5.01
E
Pyroglutamic acid
130.1 > 84.01
1.774e+006
1.49
3.06
4.27
6.29
F
Deoxyguanosine
268.1 > 152.1
9.120e+005
2.89
G
Guanosine
284.1 > 152.1
2.019e+006
2.20
H
Guanine
152 > 135
3.686e+005
1.67
2.20
2.89
3.07
4.27
6.28
100
%
0
min
1.00
2.00
3.00
4.00
5.00
6.00
