# Supplementary material for: Metabolomic Differences Between Wild and Cultivated Ophiocordyceps sinensis Identify Source-Associated Metabolites with Predicted Antifibrotic Relevance
Source: Int J Mol Sci. 2026 Oct 26;27(21):9558. doi: 10.3390/ijms27219558 (PMC13763523; doi:10.3390/ijms27219558)
Supplement: Supplementary file 1 [file ijms-27-09558-s001.zip › Supplementary Figure S4.pdf]

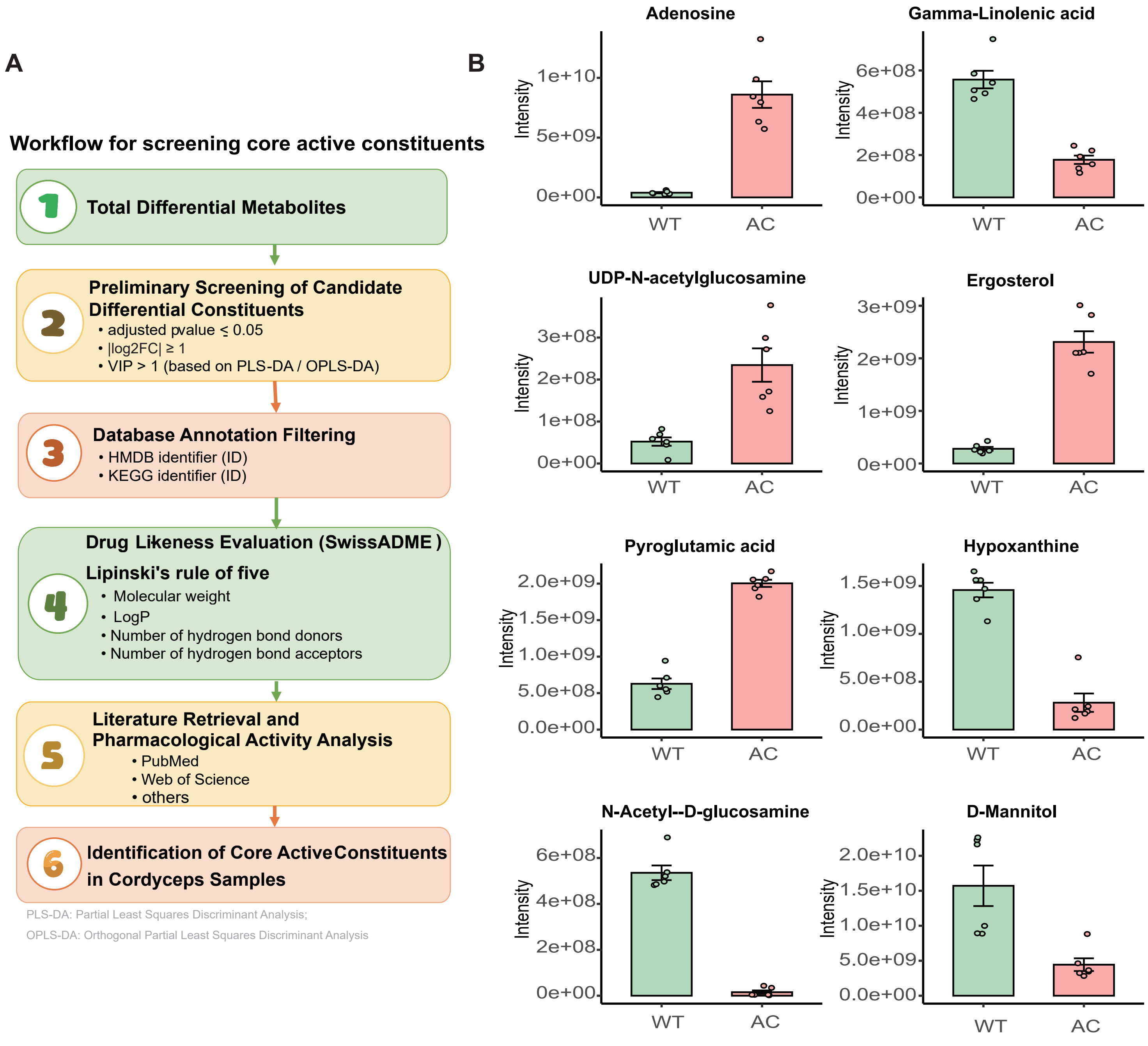
A
Workflow for screening core active constituents
1 Total Differential Metabolites
2 Preliminary Screening of Candidate Differential Constituents
• adjusted pvalue ≤ 0.05
• |log2FC| ≥ 1
• VIP > 1 (based on PLS-DA / OPLS-DA)
3 Database Annotation Filtering
• HMDB identifier (ID)
• KEGG identifier (ID)
4 Drug Likeness Evaluation (SwissADME)
Lipinski's rule of five
• Molecular weight
• LogP
• Number of hydrogen bond donors
• Number of hydrogen bond acceptors
5 Literature Retrieval and Pharmacological Activity Analysis
• PubMed
• Web of Science
• others
6 Identification of Core Active Constituents in Cordyceps Samples
PLS-DA: Partial Least Squares Discriminant Analysis;
OPLS-DA: Orthogonal Partial Least Squares Discriminant Analysis
B
Adenosine
Gamma-Linolenic acid
UDP-N-acetylglucosamine
Ergosterol
Pyroglutamic acid
Hypoxanthine
N-Acetyl--D-glucosamine
D-Mannitol
Intensity
WT
AC
