# Supplementary material for: Metabolomic Differences Between Wild and Cultivated Ophiocordyceps sinensis Identify Source-Associated Metabolites with Predicted Antifibrotic Relevance
Source: Int J Mol Sci. 2026 Oct 26;27(21):9558. doi: 10.3390/ijms27219558 (PMC13763523; doi:10.3390/ijms27219558)
Supplement: Supplementary file 1 [file ijms-27-09558-s001.zip › Supplementary Figure S5.pdf]

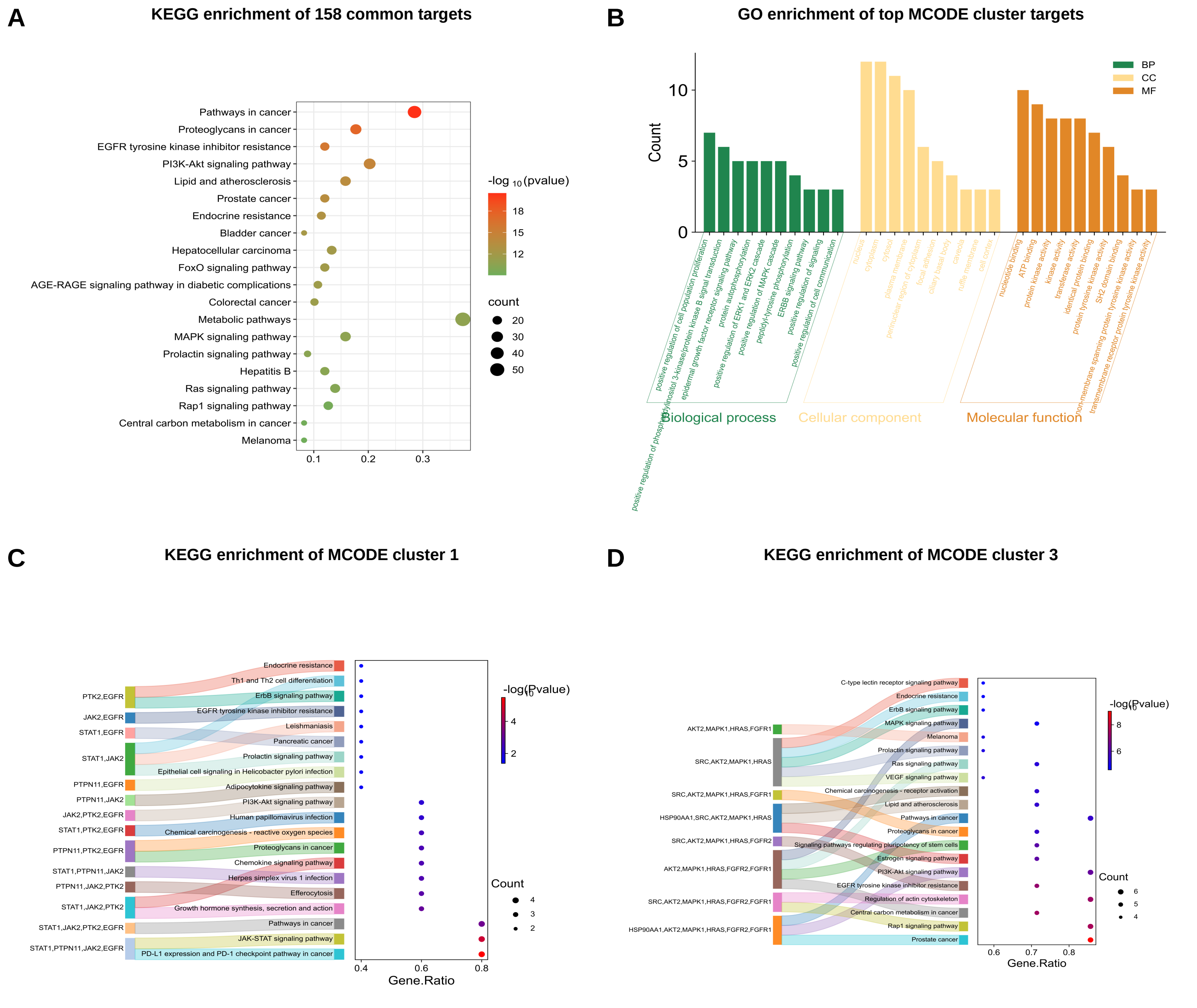
A
KEGG enrichment of 158 common targets
Pathways in cancer
Proteoglycans in cancer
EGFR tyrosine kinase inhibitor resistance
PI3K-Akt signaling pathway
Lipid and atherosclerosis
Prostate cancer
Endocrine resistance
Bladder cancer
Hepatocellular carcinoma
FoxO signaling pathway
AGE-RAGE signaling pathway in diabetic complications
Colorectal cancer
Metabolic pathways
MAPK signaling pathway
Prolactin signaling pathway
Hepatitis B
Ras signaling pathway
Rap1 signaling pathway
Central carbon metabolism in cancer
Melanoma
0.1
0.2
0.3
-log 10 (pvalue)
18
15
12
count
20
30
40
50
B
GO enrichment of top MCODE cluster targets
Count
10
5
0
BP
CC
MF
positive regulation of cell population proliferation
positive regulation of phosphatidylinositol 3-kinase/protein kinase B signal transduction
epidermal growth factor receptor signaling pathway
protein autophosphorylation
positive regulation of ERK1 and ERK2 cascade
positive regulation of MAPK cascade
peptidyl-tyrosine phosphorylation
ERBB signaling pathway
positive regulation of signaling
positive regulation of cell communication
nucleus
cytoplasm
cytosol
plasma membrane
perinuclear region of cytoplasm
focal adhesion
ciliary basal body
caveola
ruffle membrane
cell cortex
nucleotide binding
ATP binding
protein kinase activity
kinase activity
transferase activity
identical protein binding
protein tyrosine kinase activity
SH2 domain binding
non-membrane spanning protein tyrosine kinase activity
transmembrane receptor protein tyrosine kinase activity
Biological process
Cellular component
Molecular function
C
KEGG enrichment of MCODE cluster 1
PTK2,EGFR
JAK2,EGFR
STAT1,EGFR
STAT1,JAK2
PTPN11,EGFR
PTPN11,JAK2
JAK2,PTK2,EGFR
STAT1,PTK2,EGFR
PTPN11,PTK2,EGFR
STAT1,PTPN11,JAK2
PTPN11,JAK2,PTK2
STAT1,JAK2,PTK2
STAT1,JAK2,PTK2,EGFR
STAT1,PTPN11,JAK2,EGFR
Endocrine resistance
Th1 and Th2 cell differentiation
ErbB signaling pathway
EGFR tyrosine kinase inhibitor resistance
Leishmaniasis
Pancreatic cancer
Prolactin signaling pathway
Epithelial cell signaling in Helicobacter pylori infection
Adipocytokine signaling pathway
PI3K-Akt signaling pathway
Human papillomavirus infection
Chemical carcinogenesis - reactive oxygen species
Proteoglycans in cancer
Chemokine signaling pathway
Herpes simplex virus 1 infection
Efferocytosis
Growth hormone synthesis, secretion and action
Pathways in cancer
JAK-STAT signaling pathway
PD-L1 expression and PD-1 checkpoint pathway in cancer
0.4
0.6
0.8
Gene.Ratio
-log10(Pvalue)
4
2
Count
4
3
2
D
KEGG enrichment of MCODE cluster 3
AKT2,MAPK1,HRAS,FGFR1
SRC,AKT2,MAPK1,HRAS
SRC,AKT2,MAPK1,HRAS,FGFR1
HSP90AA1,SRC,AKT2,MAPK1,HRAS
SRC,AKT2,MAPK1,HRAS,FGFR2
AKT2,MAPK1,HRAS,FGFR2,FGFR1
SRC,AKT2,MAPK1,HRAS,FGFR2,FGFR1
HSP90AA1,AKT2,MAPK1,HRAS,FGFR2,FGFR1
C-type lectin receptor signaling pathway
Endocrine resistance
ErbB signaling pathway
MAPK signaling pathway
Melanoma
Prolactin signaling pathway
Ras signaling pathway
VEGF signaling pathway
Chemical carcinogenesis - receptor activation
Lipid and atherosclerosis
Pathways in cancer
Proteoglycans in cancer
Signaling pathways regulating pluripotency of stem cells
Estrogen signaling pathway
PI3K-Akt signaling pathway
EGFR tyrosine kinase inhibitor resistance
Regulation of actin cytoskeleton
Central carbon metabolism in cancer
Rap1 signaling pathway
Prostate cancer
0.6
0.7
0.8
Gene.Ratio
-log10(Pvalue)
8
6
Count
6
5
4
