# Supplementary material for: Metabolomic Differences Between Wild and Cultivated Ophiocordyceps sinensis Identify Source-Associated Metabolites with Predicted Antifibrotic Relevance
Source: Int J Mol Sci. 2026 Oct 26;27(21):9558. doi: 10.3390/ijms27219558 (PMC13763523; doi:10.3390/ijms27219558)
Supplement: Supplementary file 1 [file ijms-27-09558-s001.zip › Supplementary Figure S6.pdf]

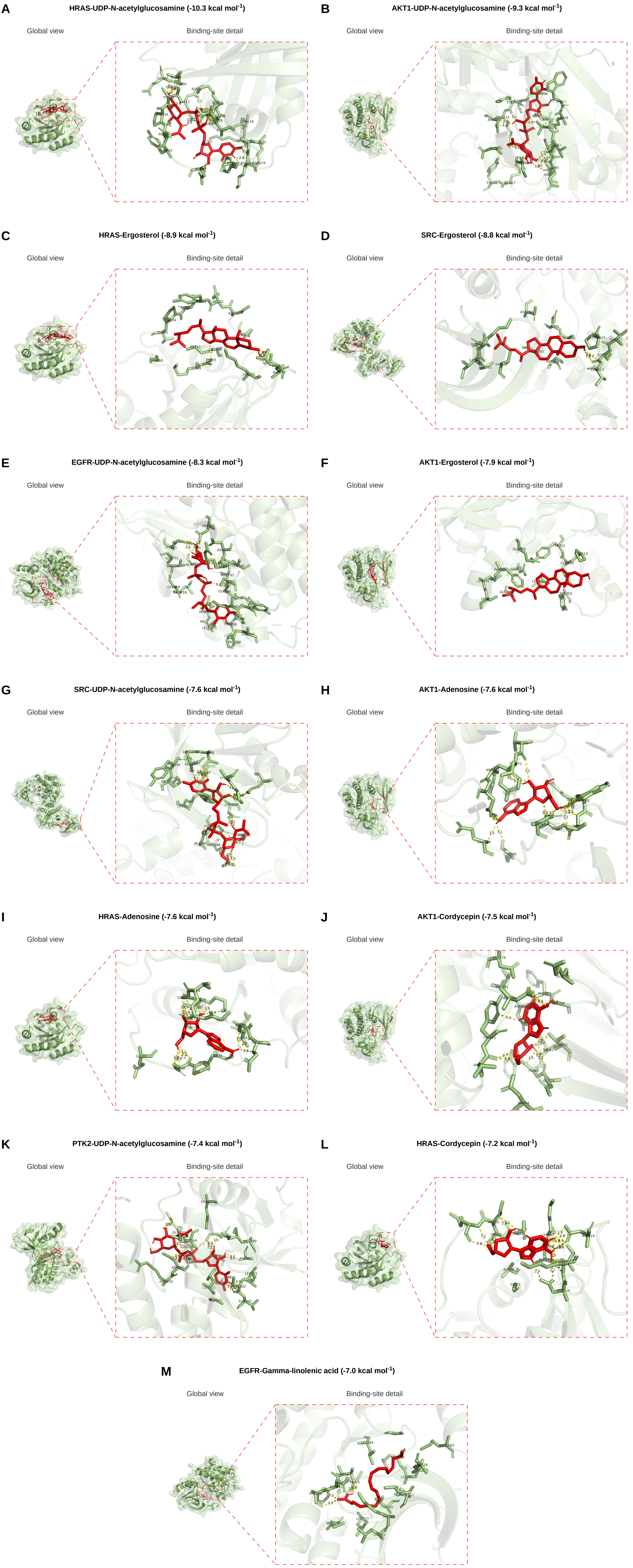
A
HRAS-UDP-N-acetylglucosamine (-10.3 kcal mol-1)
Global view
Binding-site detail
B
AKT1-UDP-N-acetylglucosamine (-9.3 kcal mol-1)
Global view
Binding-site detail
C
HRAS-Ergosterol (-8.9 kcal mol-1)
Global view
Binding-site detail
D
SRC-Ergosterol (-8.8 kcal mol-1)
Global view
Binding-site detail
E
EGFR-UDP-N-acetylglucosamine (-8.3 kcal mol-1)
Global view
Binding-site detail
F
AKT1-Ergosterol (-7.9 kcal mol-1)
Global view
Binding-site detail
G
SRC-UDP-N-acetylglucosamine (-7.6 kcal mol-1)
Global view
Binding-site detail
H
AKT1-Adenosine (-7.6 kcal mol-1)
Global view
Binding-site detail
I
HRAS-Adenosine (-7.6 kcal mol-1)
Global view
Binding-site detail
J
AKT1-Cordycepin (-7.5 kcal mol-1)
Global view
Binding-site detail
K
PTK2-UDP-N-acetylglucosamine (-7.4 kcal mol-1)
Global view
Binding-site detail
L
HRAS-Cordycepin (-7.2 kcal mol-1)
Global view
Binding-site detail
M
EGFR-Gamma-linolenic acid (-7.0 kcal mol-1)
Global view
Binding-site detail
