# Supplementary material for: Metabolomic Differences Between Wild and Cultivated Ophiocordyceps sinensis Identify Source-Associated Metabolites with Predicted Antifibrotic Relevance
Source: Int J Mol Sci. 2026 Oct 26;27(21):9558. doi: 10.3390/ijms27219558 (PMC13763523; doi:10.3390/ijms27219558)
Supplement: Supplementary file 1 [file ijms-27-09558-s001.zip › Supplementary Table S2.pdf]

Supplementary Table S2 Standard curve parameters and quantitative contents of eight selected metabolites in between AC and WT *O. sinensis*

Compound	RT (min)	Sample Source	Calibration range (ng/mL)	Regression equation	R^2	Content (μg/g DW)
Adenosine	2.62	WT	0.1-1000ng	y=5325.39*x+199.824	0.999061	3.63 ± 1.98
		AC				15.24 ± 5.02
Cordycepin	3.11	WT	0.1-1000ng	y=5153.77*x+2010.33	0.99958	3.53 ± 0.53
		AC				4.93 ± 0.72
Ergosterol	4.09	WT	0.1-1000ng	y=831.512*x+13771.3	0.975467	118.04 ± 4.76
		AC				278.84 ± 8.51
Hypoxanthine	1.84	WT	0.1-1000ng	y=338.209*x+506.949	0.998967	62.88 ± 33.58
		AC				16.57 ± 6.11
Pyroglutamic acid	1.49	WT	0.1-1000ng	y=1584.85*x+4447.81	0.998103	399.89 ± 111.70
		AC				510.81 ± 105.00
Deoxyguanosine	2.89	WT	0.1-1000ng	y=944.139*x+125.45	0.999885	29.93 ± 1.97
		AC				8.52 ± 2.97
Guanosine	2.2	WT	0.1-1000ng	y=1628.58*x+909.54	0.999818	304.38 ± 34.42
		AC				186.40 ± 18.18
Guanine	1.67	WT	0.1-1000ng	y=235.98*x+300.17	0.999053	82.20 ± 26.58
		AC				17.65 ± 7.06
