# Supplementary material for: Metabolomic Differences Between Wild and Cultivated Ophiocordyceps sinensis Identify Source-Associated Metabolites with Predicted Antifibrotic Relevance
Source: Int J Mol Sci. 2026 Oct 26;27(21):9558. doi: 10.3390/ijms27219558 (PMC13763523; doi:10.3390/ijms27219558)
Supplement: Supplementary file 1 [file ijms-27-09558-s001.zip › Supplementary Table S3.pdf]

Supplementary Table S3. Selected candidate metabolites for further analysis in AC and WT O. sinensis

Name	Class	log_2FC	p value	FDR	VIP	score	Direction（WT vs. AC)
Adenosine	Nucleosides	-4.454	8.31×10^{-9}	2.76×10^{-6}	1.711	91.7	Down
Gamma-linolenic acid	Polyunsaturated fatty acid	1.647	1.99×10^{-5}	0.000378	1.634	89.1	Up
UDP-N-acetylglucosamine	Nucleotide sugar	-2.168	0.001974	0.011325	1.423	98.7	Down
Pyroglutamic acid	Amino acid derivative	-1.674	6.42×10^{-5}	0.000891	1.669	93	Down
Hypoxanthine	Purine derivative	2.374	0.000619	0.004847	1.585	94.2	Up
N-Acetyl-D-glucosamine	Amino sugar	5.121	0.000441	0.003778	1.615	92.6	Up
Ergosterol	Sterol	-3.046	1.40×10^{-7}	1.55×10^{-5}	1.698	93.8	Down
D-Mannitol	Sugar alcohol	1.824	0.000726	0.004234	1.366	98.9	Up

Note: Direction indicates the relative abundance in WT compared with AC. FC, fold change; FDR, false discovery rate; VIP, variable importance in projection; UDP, uridine diphosphate.
