# Supplementary material for: Metabolomic Differences Between Wild and Cultivated Ophiocordyceps sinensis Identify Source-Associated Metabolites with Predicted Antifibrotic Relevance
Source: Int J Mol Sci. 2026 Oct 26;27(21):9558. doi: 10.3390/ijms27219558 (PMC13763523; doi:10.3390/ijms27219558)
Supplement: Supplementary file 1 [file ijms-27-09558-s001.zip › Supplementary-Method-S1.pdf]

Supplementary Method S1 Detailed experimental procedures

1 Untargeted metabolomics analysis

Chromatographic conditions Ultra-performance liquid chromatography (UPLC) separation was performed on an was performed on an ACQUITY UPLC HSS T3 column (100 Å, 1.8 µm, 2.1 mm × 100 mm; Waters, Milford, MA, USA) maintained at 40 °C. The autosampler temperature was set to 8 °C, and the injection volume was 2 µL. The mobile phase consisted of (A) 0.1% (v/v) formic acid in water and (B) 0.1% (v/v) formic acid in acetonitrile. A flow rate of 0.4 mL/min was used with the following gradient program: 0–1 min, 5% B; 1–4.7 min, 5% to 95% B; 4.7–6 min, 95% B; 6–6.1 min, 95% to 5% B; 6.1–8.5 min, 5% B.

Mass spectrometry conditions Mass spectrometric analysis was conducted on a Thermo Orbitrap Exploris 120 instrument (Thermo Fisher Scientific) equipped with a heated electrospray ionization (HESI) source and controlled by Xcalibur software (version 4.7). Data were acquired in data-dependent acquisition (DDA) mode, alternating between positive and negative ion modes. The HESI parameters were as follows: spray voltage, +3.5 kV/–3.0 kV; sheath gas flow, 40 arbitrary units (arb); auxiliary gas flow, 10 arb; capillary temperature, 320 °C; auxiliary gas heater temperature, 300 °C. Full-scan mass spectrometry (MS) spectra spectra were recorded at a resolution of 60,000 over the mass range m/z 70–1000, with an automatic gain control (AGC) target set to "standard" and a maximum injection time (Max IT) of 100 ms. The top four most intense ions from each full scan were selected for tandem mass spectrometry (MS/MS) with a dynamic exclusion of 4 s. MS/MS spectra were acquired at a resolution of 15,000 using higher-energy collisional dissociation (HCD) with a normalized collision energy of 30%; the AGC target was set to "standard" and Max IT to "auto."

Quality control All experimental and quality control (QC) samples were analyzed under the chromatographic and mass spectrometric conditions described above. Prior to the analytical sequence, 2–4 injections of a pooled QC sample were performed to equilibrate the system. During the sequence, one QC sample was injected after every 5–10 experimental samples to monitor instrument stability. For sample sets containing five or fewer samples, no QC injections were interspersed; these data were evaluated separately for subsequent quality assessment.

The raw mass spectrometry data were imported for peak extraction, peak alignment,

peak filtering, and metabolite annotation. Chromatographic peaks that were not detected in more than 50% of QC samples were removed. Missing values were then imputed using the built-in gap-filling algorithm, and peak areas were normalized prior to downstream analysis. Metabolite identification was performed using the PerSonalbio Next-Generation Metabolomics Database (PSNGM). The main parameters were set as follows: MS1 mass tolerance, 0.01 Da; MS2 mass tolerance, 0.05 Da; smoothing level, 3; minimum peak height, 10,000; minimum peak width, 5; mass slice width, 0.05; and identification score threshold, 60.

Based on the identified differential metabolites, core active constituents were further screened according to previously reported strategies. Candidate metabolites were selected using the following criteria: adjusted p value < 0.05, $|\log_2$ fold change (FC)$|$ ≥ 1, and variable importance in projection (VIP) > 1 derived from the partial least squares discriminant analysis (PLS-DA) or orthogonal partial least squares discriminant analysis (OPLS-DA) model. In addition, candidate metabolites were required to have both Human Metabolome Database (HMDB) and Kyoto Encyclopedia of Genes and Genomes (KEGG) identifiers.

2 Targeted quantification of selected metabolites by ultra-performance liquid chromatography–tandem mass spectrometry (UPLC–MS/MS)

Targeted liquid chromatography – tandem mass spectrometry (LC – MS/MS) analysis was performed to quantify eight selected metabolites, including adenosine, cordycepin, ergosterol, hypoxanthine, pyroglutamic acid, deoxyguanosine, guanosine, and guanine, in wild type (WT) and artificially cultivated (AC) *O. sinensis*. An independent set of five biological replicates per group was used, with each replicate representing an individual specimen.

For sample preparation, approximately 50 mg of dried, finely powdered material was extracted with 1 mL of 70% (v/v) aqueous methanol. The mixture was vortexed for 30 – 60 s and sonicated for 30 min at 40 kHz while maintaining the temperature below 30 °C. Following a 10 min equilibration at room temperature, the samples were centrifuged at 12,000 rpm for 10 min at 4 °C. The resulting supernatants were collected, passed through 0.22 μm membrane filters, and diluted 50-fold with the extraction solvent before LC – MS/MS analysis.

Eight selected metabolites, including adenosine, cordycepin, ergosterol, hypoxanthine, pyroglutamic acid, deoxyguanosine, guanosine, and guanine, were

subjected to targeted quantitative analysis. Authentic reference standards of each analyte were serially diluted to prepare calibration solutions at different concentrations. External calibration curves were constructed by plotting the chromatographic peak response against the corresponding standard concentration, and metabolite concentrations in the samples were calculated using the respective calibration equations.

Targeted LC–MS/MS analysis was performed using an ACQUITY UPLC Premier system coupled to a Xevo TQ-Absolute triple-quadrupole mass spectrometer (Waters, Milford, MA, USA). Chromatographic separation was achieved on an ACQUITY UPLC BEH C18 column (100 × 2.1 mm, 1.7 μm; Waters) maintained at 45 °C. The mobile phases consisted of water containing 0.1% formic acid (A) and acetonitrile containing 0.1% formic acid (B), with a linear gradient from 1% to 100% B. The flow rate was 0.4 mL/min, and the injection volume was 1 μL.

The mass spectrometer was operated in multiple reaction monitoring (MRM) mode with positive electrospray ionization. The capillary voltage was set at 1 kV, the source temperature at 150 °C, and the desolvation temperature at 450 °C. The desolvation and cone gas flow rates were 900 and 50 L/h, respectively. Nitrogen (>99.5%) was used as the desolvation and cone gas, and argon (>99.999%) was used as the collision gas. The scan time was 0.01 s per transition. The optimized MRM transitions were as follows: guanosine, m/z 284.1 → 152.1 (cone voltage, 20 V; collision energy, 30 eV); adenosine, m/z 268.1 → 136.2 (20 V; 25 eV); guanine, m/z 152.2 → 135.3 (30 V; 25 eV); deoxyguanosine, m/z 268.1 → 152.2 (20 V; 20 eV); ergosterol, m/z 379.3 → 69.3 (30 V; 25 eV); pyroglutamic acid, m/z 130.2 → 84.2 (20 V; 25 eV); cordycepin, m/z 252.1 → 136.2 (30 V; 30 eV); and hypoxanthine, m/z 137.2 → 119.3 (20 V; 30 eV). Data acquisition and processing were performed using MassLynx 4.2 software (Waters).

3 Molecular docking

Molecular docking between candidate bioactive constituents of *O. sinensis* and AKT serine/threonine kinase 1 (AKT1; Protein Data Bank [PDB] ID: 3O96),, HRas proto-oncogene, GTPase (HRAS; PDB ID: 5P21) , epidermal growth factor receptor (EGFR; PDB ID: 1XKK), mitogen-activated protein kinase 1 (MAPK1; PDB ID:

1TVO), protein tyrosine kinase 2 (PTK2; PDB ID: 4I4E), and proto-oncogene tyrosine-protein kinase Src (SRC; PDB ID: 4MXO) was performed using AutoDock Vina version 1.2.0 with the Vina scoring function [51,52]. Receptor and ligand PDBQT (PDB coordinates with partial charges and atom types) files were prepared using the prepare_receptor4.py and prepare_ligand4.py scripts implemented in AutoDockTools (MGLTools version 1.5.7) with default settings. During PDBQT preparation, polar hydrogen atoms were retained, nonpolar hydrogen atoms were merged, and Gasteiger partial charges were assigned.

Three representative protein–ligand complexes were selected for subsequent molecular dynamics (MD) simulations on the basis of their docking scores, target relevance within the predicted signaling network, and ligand diversity: HRAS – uridine diphosphate N-acetylglucosamine (UDP-N-acetylglucosamine), and SRC–ergosterol. Representative low-energy docking poses were used as the initial structures for the MD simulations. For ligand parameterization, electrostatic potentials were calculated at the Hartree – Fock (HF)/6-31G(d) level, followed by two-stage restrained electrostatic potential (RESP) charge fitting according to the established RESP procedure. Bonded and nonbonded force-field parameters for the ligands were generated using Antechamber in the AmberTools suite [53].

All MD simulations were performed using the graphics processing unit (GPU)-accelerated pmemd.CUDA engine in Amber 22 with an integration time step of 2 fs [54]. Each protein–ligand complex was solvated in a cubic box of three-site transferable intermolecular potential (TIP3P) water water [55], with a minimum distance of 10 Å between the solute and the box boundary. Counterions were added to neutralize each system, and periodic boundary conditions were applied in all three dimensions.

Each solvated complex was subjected to a two-stage energy-minimization protocol. In the first stage, solvent molecules and counterions were minimized while positional restraints were applied to the heavy atoms of the protein and ligand. In the second stage, the entire system was minimized without positional restraints to eliminate unfavorable contacts. The minimized systems were then gradually heated from 0 to 300 K over 100 ps using a Langevin thermostat, followed by sequential equilibration under the constant-number, constant-volume, constant-temperature (NVT) and constant-number, constant-pressure, constant-temperature (NPT) ensembles at 300 K and 1 bar. Pressure was controlled using a Berendsen barostat

during equilibration.

To assess reproducibility and reduce dependence on any single trajectory, three independent MD simulations were performed for each protein–ligand complex. Each production trajectory was initially propagated for 100 ns at 300 K and 1 bar. Trajectories that maintained stable and representative ligand-binding conformations were subsequently extended to 500 ns for further characterization. Trajectory processing and structural analyses were performed using CPPTRAJ [56], and representative conformations were extracted by cluster analysis for subsequent interaction analyses. Noncovalent interactions were further characterized using the independent gradient model based on Hirshfeld partitioning (IGMH) [23] and topological analysis within the atoms-in-molecules (AIM) framework. Wavefunction-based analyses and visualization were performed using Multiwfn [57].

References

51. Kouranov A, Xie L, de la Cruz J, Chen L, Westbrook J, Bourne PE, et al. The RCSB PDB information portal for structural genomics. Nucleic Acids Res. 2006;34(Database issue):D302–D305. https://doi.org/10.1093/nar/gkj120.

52. Eberhardt J, Santos-Martins D, Tillack AF, Forli S. AutoDock Vina 1.2.0: new docking methods, expanded force field, and Python bindings. J Chem Inf Model. 2021;61(8):3891–3898. https://doi.org/10.1021/acs.jcim.1c00203.

53. Case DA, Aktulga HM, Belfon K, Cerutti DS, Cisneros GA, Cruzeiro VWD, et al. AmberTools. J Chem Inf Model. 2023;63(20):6183–6191. https://doi.org/10.1021/acs.jcim.3c01153.

54. Salomon-Ferrer R, Götz AW, Poole D, Le Grand S, Walker RC. Routine microsecond molecular dynamics simulations with AMBER on GPUs. 2. Explicit solvent particle mesh Ewald. J Chem Theory Comput. 2013;9(9):3878–3888. https://doi.org/10.1021/ct400314y.

55. Jorgensen WL, Chandrasekhar J, Madura JD, Impey RW, Klein ML. Comparison of simple potential functions for simulating liquid water. J Chem Phys. 1983;79(2):926–935. https://doi.org/10.1063/1.445869.

56. Roe DR, Cheatham TE III. PTRAJ and CPPTRAJ: software for processing and analysis of molecular dynamics trajectory data. J Chem Theory Comput. 2013;9(7):3084–3095. https://doi.org/10.1021/ct400341p.

23. Lu T, Chen Q. Independent gradient model based on Hirshfeld partition: a new method for visual study of interactions in chemical systems. J Comput Chem. 2022;43(8):539–555. https://doi.org/10.1002/jcc.26812.

57. Lu T, Chen F. Multiwfn: a multifunctional wavefunction analyzer. J Comput Chem. 2012;33(5):580–592. https://doi.org/10.1002/jcc.22885.
